# Large‐scale experimental evaluation of woody plant removal in desert grassland: Restoration, novelty, or degradation?

**DOI:** 10.1002/eap.70240

**Published:** 2026-04-21

**Authors:** Brandon T. Bestelmeyer, Laura M. Burkett, Darren James, Juan Gamon, Robert L. Schooley

**Affiliations:** ^1^ United States Department of Agriculture, Agricultural Research Service Jornada Experimental Range Las Cruces New Mexico USA; ^2^ Las Cruces District Office, USDI Bureau of Land Management Las Cruces New Mexico USA; ^3^ Department of Natural Resources and Environmental Sciences University of Illinois Urbana Illinois USA

**Keywords:** grasslands; grazing, herbicide; long‐term monitoring, perennial grasses, restoration, shrub encroachment

## Abstract

Woody plant encroachment into grassy ecosystems is a worldwide phenomenon that radically transforms ecosystem services. Billions of dollars have been spent to remove woody plants, but there is great uncertainty about the conditions in which such removals are beneficial. We conducted a collaborative, large‐scale monitoring experiment in the Chihuahuan Desert of southern New Mexico, USA, to test whether woody plant removal restored historical states, created novel states, or exacerbated land degradation and to examine the environmental conditions affecting outcomes. We monitored vegetation cover in 43 pairs of plots representing herbicide‐treated and herbicide‐untreated conditions of the same plant community and environmental setting, including measurements at baseline, 5, 10, and in some cases 15 years posttreatment. We compared outcomes to measurements in reference sites. Woody plant removal led to increases in plant species richness and perennial grass cover, but increases were due to disturbance‐adapted grasses rather than species characteristic of reference states. On average, grass cover in treatments did not attain levels observed in the reference state. Negative effects of woody plant removal on total canopy cover (related to soil erosion risk) and other plant functional groups of concern for wildlife were not observed. Elevation, slope, and baseline cover were important predictors of treatment‐associated gains in plant cover, while higher grazing intensity was related to increases in richness and forb cover. Our results indicate that while woody plant removal cannot be considered to restore a reference state due to the continued absence of reference grass species, it does not lead to obvious land degradation. Furthermore, more positive outcomes are maximized where (and when) soil moisture limitations are lowest or the cover of responding plants is highest prior to treatments. We recommend that future restoration actions be conducted as experiments that pay special attention to co‐production mechanisms, standardized monitoring methods, and salient and easily measured indicators.

## INTRODUCTION

Woody plant encroachment into grasslands, savannas, and other ecosystems is a worldwide phenomenon that radically transforms ecosystem structure and functions (Ding & Eldridge, 2024). Encroachment is driven by a variety of conditions that favor establishment and growth of woody plants at the expense of herbaceous plants, including preferential grazing by livestock on grasses, elimination of fire disturbances that limit woody plant dominance, increasing aridity, and rising atmospheric CO_2_ levels that favor C3 woody plants over C4 grasses (Archer et al., 2017). Although the effects of encroachment into ecosystem services vary greatly by location, woody plant traits, and human perspectives (Eldridge et al., 2011; Eldridge & Ding, 2021), many observers regard woody encroachment as having negative impacts on livestock production, habitat quality for grassland‐associated species, and soil stability (Anadón et al., 2014; Archer & Predick, 2014). In such cases, woody encroachment represents a form of land degradation and a departure from historical reference conditions which triggers restorative land management actions (Archer et al., 2011). Consequently, land managers worldwide have expended billions of dollars on woody plant removal through various means, including physical removal, burning, and use of selective herbicides (Ding et al., 2020; Ding & Eldridge, 2024).

Like other restoration actions, the effectiveness of woody plant removal in achieving restoration goals is questioned for several reasons. First, there are relatively few systematic monitoring programs to evaluate removal effects over time, rather than point‐in‐time comparisons that provide an incomplete understanding of restoration effects (Briske et al., 2017; Cooke et al., 2019). Second, restoration goals are often poorly defined, unrealistic, and not collaboratively developed by scientists and stakeholders, precluding a broadly accepted understanding of what outcomes constitute success (Brudvig & Catano, 2024; Cooke et al., 2019). Third, much of our restoration evidence comes from fine‐scale studies rather than studies of operational projects in large‐scale landscapes that incorporate sources of variability in restoration applications, environmental setting, and posttreatment land management (Giardina et al., 2007; Roccaforte et al., 2024). Consequently, our ability to explain and predict variability in restoration outcomes as a basis for planning remains limited (Brudvig et al., 2017; Ding & Eldridge, 2019). Fourth, tests of restoration outcomes are seldom conducted as replicated “true” experiments that control for key sources of variability, such as initial conditions and environmental setting, and thus cannot precisely measure effects and explain variability (Brudvig et al., 2021; Fick et al., 2022).

It is also challenging to interpret variable restoration outcomes as a basis for evaluating success and making subsequent decisions (Hobbs et al., 2014; Miller & Bestelmeyer, 2016). Some cases may achieve measurable vegetation goals (e.g., a return to a reference state) and therefore be defined as “restoration.” Alternatively, historically based restoration goals may not be achieved when ecological thresholds have been crossed, but outcomes may feature unprecedented species assemblages that are nonetheless desirable considering the available options, referred to as “novel ecosystems.” Finally, restoration actions may inadvertently exacerbate degradation if there are unexpected, undesirable changes in ecosystem functions or if the reference state is incorrectly identified, for example, when woody plants are removed from historically wooded ecosystems (Eldridge & Ding, 2021; Parr et al., 2024).

Here, we employ recent advances in restoration thinking in a collaborative, large‐scale experiment on woody plant removal in the desert grassland region of the southwestern United States. The history of widespread woody encroachment in this region is well documented via repeat photography, historical records, and long‐term monitoring (Bestelmeyer et al., 2018; Christensen et al., 2023). Given the perceived negative impacts of encroachment, generous government support has been provided to landowners across the United States to manage woody plants on 0.87–1.7 million ha/year of grazing lands (since 2005) at an estimated cost of tens of millions of dollars per year (Briske et al., 2017; USDA Natural Resources Conservation Service, 2021). In the desert grassland region of New Mexico, woody plant removal via selective herbicides has occurred on approximately 14% of public lands from 1982 to 2016, most occurring in the latter years of that period (Bestelmeyer et al., 2018). Although passive vegetation management is more extensive, including grazing plans and livestock fencing, woody plant removal is the most pervasive direct manipulation of ecosystem structure and function in the western United States (Briske et al., 2017).

The benefits received from woody plant removal, however, are debated by stakeholders. In principle, removal in arid ecosystems should reduce the strong competition by woody plants for soil water resources and trigger the recovery of perennial grasses (Pierce et al., 2019) that support key ecosystem services of interest, including forage for cattle, grassland bird habitat, and erosion control (Bestelmeyer et al., 2018; Coffman et al., 2014; Macías‐Duarte et al., 2018; Webb et al., 2014). Localized studies suggest that woody plant removal may or may not result in perennial grass recovery (Brock et al., 2014; Mata‐González et al., 2007). Based on a survey of US Bureau of Land Management (BLM) staff responsible for managing restoration projects in our study area, most believed that treatments to remove encroaching desert shrubs are effective in reducing woody plant cover and increasing perennial grasses but varied in their belief that historical grassland composition and the more abstract “grassland health” was restored (Appendix S1: Figure S1). Moreover, the lack of systematic, broad‐scale monitoring underpins substantial disagreement regarding the value of removal efforts in the broader community, or even if such efforts can be considered restoration at all.

A fundamental question is: Have shrub‐encroached grasslands crossed biotic or abiotic thresholds that preclude the recovery of perennial grasses once resource competition from shrubs is reduced (Ding & Eldridge, 2023; Maestre et al., 2016; Suding & Hobbs, 2009)? The answer to this question likely depends on (1) variations in environmental context (e.g., climate, soils), requiring evaluations of restoration effects across broad spatial extents (Aoki et al., 2020; Krzywicka et al., 2017; Perring et al., 2015); (2) temporal context, especially considering increasing aridity that may limit grass recovery (Zhuang et al., 2024); and (3) the traits of shrubs and perennial grasses (Archer et al., 2011; Eldridge et al., 2011). In addition, the plant species that benefit from removal are unknown and may include functionally important “foundation species” (Ellison, 2019) characteristic of historical reference communities or weedy, transient species that produce novel communities of lower perceived value.

In this study, we embedded a long‐term monitoring program within woody plant removal treatments carried out across 2.2 million ha of public lands in southwestern New Mexico as part of the BLM‐led “Restore New Mexico” program (Bestelmeyer et al., 2019). Monitoring was designed as a true experiment and was integrated with operational treatment planning following a statistically robust “before‐after‐control‐impact” (BACI) design (Block et al., 2001). We designed and conducted our research as knowledge co‐production representing a shared research process involving scientists and stakeholders (Norström et al., 2020). Co‐production increases the likelihood that monitoring results are salient, trusted, and ultimately useful for decision‐making. Based on meetings with managers (discussed below), we identified a set of vegetation indicators and hypotheses of interest to examine the production, conservation, and sustainability goals of woody plant removal.

We tested for several predicted outcomes of woody plant removal (Table 1), including increases in species richness and the cover of high‐value perennial grasses, no change or increases in total ground cover, and the potential for negative impacts to forbs used by wildlife that represent a trade‐off with increasing grass cover. We evaluated vegetation responses in comparison to a set of reference sites within the region (Shackelford et al., 2024). We then tested the impact of environmental factors hypothesized to influence the dominance and recovery of herbaceous plants in arid systems, including soil and physiographic setting, baseline vegetation state, recent weather patterns, and grazing use (Bestelmeyer et al., 2013; Yao et al., 2006). Finally, we considered the evidence for whether shrub removal outcomes can be interpreted as restoring the reference state, creating a novel ecosystem, or exacerbating land degradation.

**TABLE 1:** Indicators of interest to stakeholders and predicted responses to woody plant removal.

Indicator	Prediction	Mechanism and rationale for importance
Species richness	Increase	Higher recruitment of species from the seed bank during favorable years due to increased resource availability
Total canopy	No change or increase	Herbaceous canopy cover increases to compensate for the loss of shrub cover. Protects soil surface from erosion.
Nontarget species	Increase	Recruitment of herbaceous and nontarget woody plants due to increased resource availability
Total perennial grasses	Increase	Perennial grass cover increases due to increased resource availability. Protects the soil surface from erosion, especially during droughts.
Reference perennial grasses	Increase	Perennial grass species characteristic of the reference state increase with increased resource availability and growing season grazing deferment. Preferred livestock forage; declines under continuous grazing.
Increaser perennial grasses	Increase	Grazing‐tolerant grasses increase when reference grasses are slow to recover. Less valuable forage; less sensitive than reference grasses to grazing pressure.
Disturbance perennial grasses	Increase	Short‐lived, shallow‐rooted ruderal grasses increase as an early successional response and persist if reference and increaser perennial grasses do not recover.
Perennial forbs	Decrease, no change, or increase	Perennial forbs may be sensitive to herbicides but may also benefit from increased resource availability. Valued as forage resources for several mammal and bird species of management interest including mule deer (*Odocoileus hemionus*), pronghorn (*Antilocapra americana*), scaled quail (*Callipepla squamata*), and various granivorous grassland birds.
Annual grasses and forbs	No change or increase	Annual grasses and forbs may increase as an early successional response and due to increased resource availability during periods of favorable rainfall.

*Note*: Indicators other than species richness refer to aspects of plant cover. Predictions refer to the effects of the treatment relative to the control. Mechanism and, where needed, the rationale for the importance of the indicator are described.

## MATERIALS AND METHODS

### Study area and treatments

The study area is in the Chihuahuan Desert of southern New Mexico, USA, in rangeland areas dominated by publicly owned land used primarily for extensive livestock production, hunting, and recreation (Appendix S1: Figure S2). Elevation ranges from 1307 to 1736 m. Mean annual temperature (MAT) ranges from 13.7°C to 16.8°C and mean annual precipitation (MAP) ranges from 238 to 297 mm, most of which falls between July and September (1981–2010, 30‐year normal, 4 km gridded data) (PRISM Climate Group, 2025). MAT during the study was similar to the 30‐year average (12.8°C–17.7°C), whereas MAP varied greatly among years, from 98 to 403 mm.

Forty‐three experimental plot pairs (a pair = one control and one treated plot) were selected using a stratified random design in 29 herbicide treatments (tebuthiuron) ranging from 168 to 10,292 ha in area. Treatment areas were selected by BLM land managers based on rancher interest and grazing allotment management objectives. Herbicide treatments were applied by aircraft at a rate of 0.56 kg/ha in nonclay soils and 0.84 kg/ha in clayey soils to shrub‐dominated plant communities featuring creosotebush (*Larrea tridentata*) with minor amounts of tarbush (*Flourensia cernua*), which are common encroaching shrub species in desert grasslands of the region (Archer et al., 2022). Plant communities historically dominated by shrubs are known to exist in the region, often on very gravelly soils with a shallow indurated soil horizon (Bestelmeyer et al., 2018; Buffington & Herbel, 1965). Such obvious “historical shrublands” were avoided in designing treatments by using soil maps (e.g., Jacobs et al., 2008). Unlike other restoration efforts involving herbicides (Munson et al., 2020), seeding of herbaceous species was not performed because such efforts have had limited success in the past (Herrick et al., 2006) and because many (but not all) desirable herbaceous species are already well represented in the seed bank (Romig et al., 2025). Posttreatment management of cattle grazing typically involved growing season rest for two or more years.

### Sampling design

In areas that were treated by BLM from 2007 to 2014, we randomly selected locations (prior to the treatments) within spatially dominant soil and vegetation types (Steele et al., 2012) in which adjacent treated and untreated (control) plot pairs were established. We aimed to include one plot pair for each 1000 ha treated such that extensive treatments received >1 plot pair. Two adjacent plots (median 261 m apart) with similar soil type, slope, aspect, and initial vegetation were established as 9‐ha (300 × 300 m) polygons delineated in ArcGIS (Esri, Redlands, CA). One member of each pair was randomly assigned as the control (Appendix S1: Figure S3). The locations of control plots were programmed into the GPS guidance systems of the aircraft applying herbicides such that controls would not receive herbicide.

Vegetation monitoring sites were established, and baseline data collected, approximately one‐year posttreatment to allow time to determine whether adequate shrub mortality occurred in response to herbicide application but before effects on nontarget plants occurred. We established monitoring only when >75% of individual targeted shrubs had >50% leaf loss or 100% leaf decadence (i.e., brown leaves). Thus, our monitoring effort focused on the vegetation response when the treatment was successful to avoid the confounding influence of variation in application efficacy (94.5% of treatment applications resulted in target shrub mortality). Following the baseline measurement, sampling occurred at 5 and 10 years posttreatment, and for 10 plots, 15 years posttreatment. Vegetation cover was estimated with the line‐point intercept method (Herrick et al., 2017) on two parallel 50‐m transects separated by 20 m with 25‐cm intervals or, in cases where 50‐m transects would cross soil or vegetation boundaries, three, 20‐m transects with 20‐cm intervals. Plants were identified at the species level when possible and placed in functional groups (Bestelmeyer et al., 2025) following the guidelines in Appendix S1: Table S1 and assignments in Appendix S2: Table S1.

Physiography and soil data were collected at the center point between transects. Soil pedons were characterized within mini pits (50 cm × 50 cm) to a 70‐cm depth or to a root restrictive horizon following Schoeneberger et al. (2012). Clay content of each soil horizon was estimated in the field using texture‐by‐feel of an experienced observer calibrated to lab‐determined soil textures using the hydrometer method. In addition, a single observer estimated grazing utilization (largely by cattle) according to two broad classes: none‐light/conservative (0%–40% removal of biomass of forage plants) and moderate‐heavy/severe (>40%) (Holechek & Galt, 2000).

### Co‐production and indicators

Indicators and hypotheses were developed in consultation with land managers at the beginning of the project in 2007–2009. The collaborative group comprised land management agencies that fund and execute restoration treatments and supporting practices (including representatives of the US Department of Agriculture Natural Resources Conservation Service, BLM, New Mexico Department of Fish and Game, among others). These efforts were coordinated with ranchers by the New Mexico Association of Soil and Water Conservation Districts (NMACD) via Coordinated Resource Management Plans. Leaders from NMACD served as “boundary spanners” that connected agencies, ranchers, and science activities (Bestelmeyer et al., 2019). In these initial meetings, we asked participants open‐ended questions including: (1) what vegetation responses do you expect or want to achieve from shrub removal, and (2) what environmental or management factors might influence those responses? From these discussions, we identified indicators of interest and expected responses summarized in Table 1 and environmental predictors summarized in Appendix S1: Table S2. While there are many approaches to evaluating change in ecosystems that are appealing to ecologists (e.g., multifunctionality; Manning et al., 2018), we focused on targeted metrics that were (a) measurable using standard methods already used by land managers, and (b) salient to managers and likely to be consulted in future management decisions and evaluation of key trade‐offs.

### Reference sites

We used measurements from multiple reference sites in pre‐project planning to provide quantitative benchmarks incorporating spatial variability to guide management goal setting and subsequent evaluation (Gann et al., 2019). Prior to initiation of the project (2005), we obtained vegetation cover records from plots in 17 reference sites using the same methods described above. Following recommendations summarized in Shackelford et al. (2024), we (1) used multiple sources of information to identify reference conditions, and (2) considered the reference sites as a practical target against which to gauge success. To designate reference sites, we first consulted information about the reference state from a state‐and‐transition model used by US federal agencies as a benchmark in rangeland health assessments (Appendix S1: Figure S4). The characteristics of the reference state in the state‐and‐transition model (high reference perennial grass cover, low shrub cover) were corroborated by a unique historical photo pair from a known location on the Jornada Experimental Range within the study region (Appendix S1: Figure S5). Reference sites expressing these historical characteristics were found in the northern portion of the study area (Appendix S1: Figure S2). Sites were judged to exhibit historical characteristics if they were dominated by reference grasses (Table 1) and had low encroaching shrub cover (<10%). Possible reasons that these sites had not undergone shrub encroachment include histories of conservative grazing management and their occurrence in the relatively wet microclimates at the northern extent of the study area. However, median elevation (which we found below to be an important determinant of restoration success) of the reference sites was close to the median of the experimental plot pairs (Appendix S1: Figure S6).

Taken together, this evidence suggests that our reference sites represent reasonable benchmarks for developing and evaluating restoration goals, recognizing that these sites are spatially clustered and are a potentially biased representation of pre‐encroachment states at the experimental sites. Variations in recent climate, elevation, subtle soil differences, and land use legacies produced variation in vegetation that we considered to represent realistic variability in reference conditions (Shackelford et al., 2024).

### Data manipulation and statistical models

We used R version 4.4.1 for all data manipulation, figure creation, and analyses (R Core Team, 2024; Wickham et al., 2019) with the exception of the linear mixed models that were fit with the MIXED procedure in SAS 9.4 (SAS Institute Inc., Cary, NC, USA). All data discussed below are available in Bestelmeyer et al. (2025).

To evaluate treatment effects over 5‐ and 10‐year periods, we examined 86 plots (43 pairs) for responses by each plant functional group and species richness. Each response was modeled separately with linear mixed‐effects models. Treatment versus control, time (baseline, 5, and 10 years), and their interaction were fixed effects, and the plot pair was a random effect. Time was modeled with an “unstructured” covariance structure. A Kenward–Roger adjustment was applied to the denominator df. We used custom contrasts to estimate model‐based gain scores at 5 and 10 years and test whether these differed from zero at 0.10, 0.05, and 0.01 alpha levels. The “gain score” measures the change in response variable from baseline (i) to each subsequent sampling period for treatments (*T*) relative to changes in the paired controls (*C*), where

$$\text{Gain score}=\left(\text{Cover}{\left(T\right)}_{5,10y}-\text{Cover}{\left(T\right)}_{i}\right)-\left(\text{Cover}{\left(C\right)}_{5,10y}-\text{Cover}{\left(C\right)}_{i}\right).$$

Thus, for example, a positive gain score may reflect greater cover increases in treatments relative to controls or a greater rate of decline in controls relative to treatments.

We also examined total canopy cover and cover of total perennial grasses, reference perennial grasses, and encroaching shrubs in 10 plot pairs with 15 years of monitoring record as above, except that time was implemented as the year of sampling (2007, 2012–2013, 2017, 2022–2023). Gain scores were not calculated; instead, we compared means of treatments, controls, and their sampling periods via least significant difference tests (alpha = 0.05).

To visualize growing season (July–September) climatic conditions during our sampling periods to help interpret treatment effects, we downloaded monthly standardized precipitation evapotranspiration index (SPEI) estimates for 1990–2024 (Abatzoglou et al., 2017), extracted data for the 86 plot locations, and then calculated annual means.

We compared total canopy cover, total perennial grasses, reference perennial grasses, and shrubs from 86 plots (treatments and controls) across sampling periods to our population of 17 reference plots measured a single time. We fitted linear models as one‐way ANOVAs with seven levels (reference and then treatment and controls at baseline, 5, and 10 years). We used Dunnett's test to compare treatment and control plot samples to the reference set (Lenth, 2025).

To examine the relative importance of environmental factors (Appendix S1: Table S2) influencing treatment effects at each sampling period, we calculated gain scores and built linear mixed‐effects models for species richness and the plant functional groups above, except for shrubs that were directly affected by treatments. In the first stage, we identified the best fitting precipitation variable (Appendix S1: Table S3) for each plant functional group and sampling period based on corrected Akaike information criterion (AICc), reasoning that different groups may respond most strongly to different timescales of precipitation (Ogle & Reynolds, 2004). We considered six potential precipitation anomaly variables: difference from 30‐year (1994–2024) mean of water year (November 1–October 31) precipitation, difference from 30‐year mean of growing season (July + August + September total) precipitation, the 1‐year lag (prior year) of each of these responses, and the average of the current and 1‐year lag of these values. Monthly precipitation rasters were obtained using the PRISM R package (Hart & Bell, 2015). Each candidate model set included seven models: one for each precipitation variable in addition to an intercept‐only model. After scaling all variables to a mean of 0 and SD of 1, we fit each model and assessed its AICc. For each of the 24 response * sampling period combinations, we chose the precipitation variable with the lowest AICc value to enter the second modeling stage (Appendix S1: Table S4) unless the intercept‐only model was within 2 AICc units of it, in which case we dropped precipitation from further consideration.

For the second stage, we considered the following candidate variables: precipitation (if retained from stage 1), elevation, slope, maximum soil clay content, baseline cover, and encroaching shrub cover (Appendix S1: Table S2). Encroaching shrub cover was omitted as a predictor of total canopy cover because shrub cover was a part of that response. For all the other responses, we examined bivariate Pearson correlations and imposed a threshold of +/− 0.40 to avoid multicollinearity. In cases where the Pearson correlation between baseline cover and/or precipitation with any other candidate predictor exceeded this threshold, we dropped these variables in favor of physiographic variables that are readily assessed using maps. We constructed the candidate model set using all possible combinations of its final predictors in addition to an intercept‐only model. After scaling the data, we fit each model and ranked them according to ΔAICc. For model sets in which the intercept‐only model was within 2 ΔAICc units of the top model, we concluded that the candidate predictors had no appreciable effect on the gain scores. For all others, we calculated AICc‐weighted standardized parameter estimates (Mazerolle, 2023).

To provide a plant community perspective on effects of woody plant removal, we conducted multiresponse permutation procedures (MRPP) with the cover values of nine functional groups that (1) included functional groups not considered in previous hypotheses, (2) grouped bunchgrasses, and (3) treated the very common *Dasyochloa pulchella* as a separate group (Appendix S2). We used Euclidian distance separately at each sampling period to compare treatment and control plots, using the “vegan” R package (Oksanen et al., 2025).

## RESULTS

**FIGURE 1:**
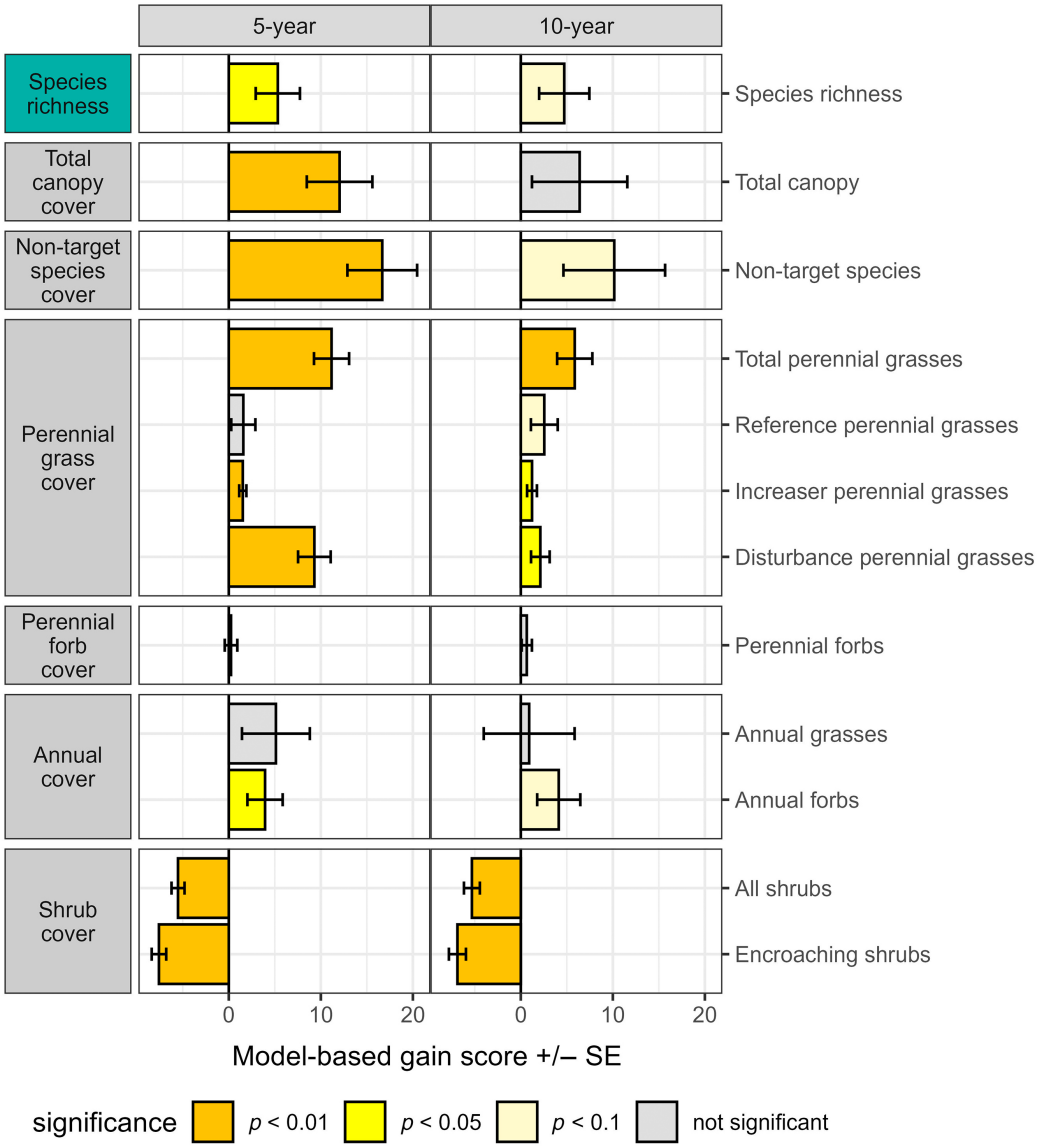
Gain score estimates (mean ± SE) for species richness and cover of plant functional groups in response to woody plant removal at 5 and 10 years posttreatment. Gain scores measure the change in a response variable from baseline to each subsequent sampling period for treatments relative to changes in the paired controls. Labels at left are major plant functional group categories and at right are specific functional groups.

Woody plant removal treatments had substantial and long‐lasting effects on species richness and the cover of plant functional groups (Figure 1). The greater species richness relative to controls was sustained for 10 years at the 0.10 alpha level. There was a substantial increase in total canopy cover after 5 years, despite loss of shrub cover, but this increase disappeared after 10 years. Total perennial grass cover increases were sustained for 10 years, but the magnitude of increase declined between 5 and 10 years. Disturbance‐adapted grasses, particularly the shallow‐rooted *D. pulchella*, were the most important components of the perennial grass response, but grazing‐tolerant “increaser” grasses showed modest increases, and the high‐value reference grasses only revealed modest increases after 10 years. Perennial forbs, for which there was concern for declines in response to herbicide use, showed no changes. Annual forbs revealed sustained increases, whereas annual grasses did not. Decreases in total shrubs and targeted encroaching shrubs were sustained over 10 years, suggesting that shrub recovery had been minimal.

**FIGURE 2:**
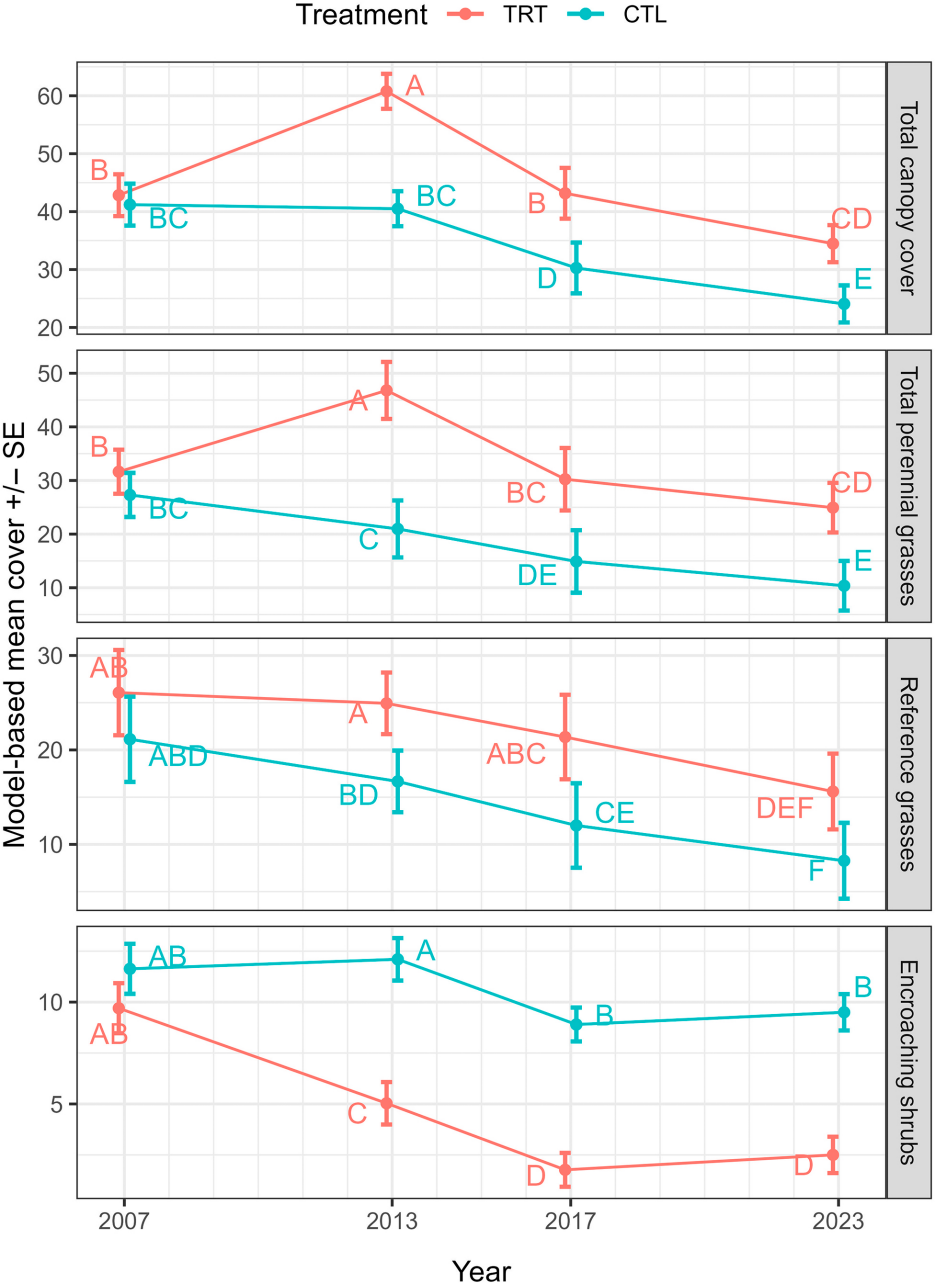
Cover (mean ± SE) of select plant functional groups in treated (TRT) and control (CTL) plots in the subset of our data with a 15‐year monitoring duration. Within a functional group, different letters indicate significant differences (*p* ≤ 0.05). Type III test results are provided in Appendix S1: Table S5.

**FIGURE 3:**
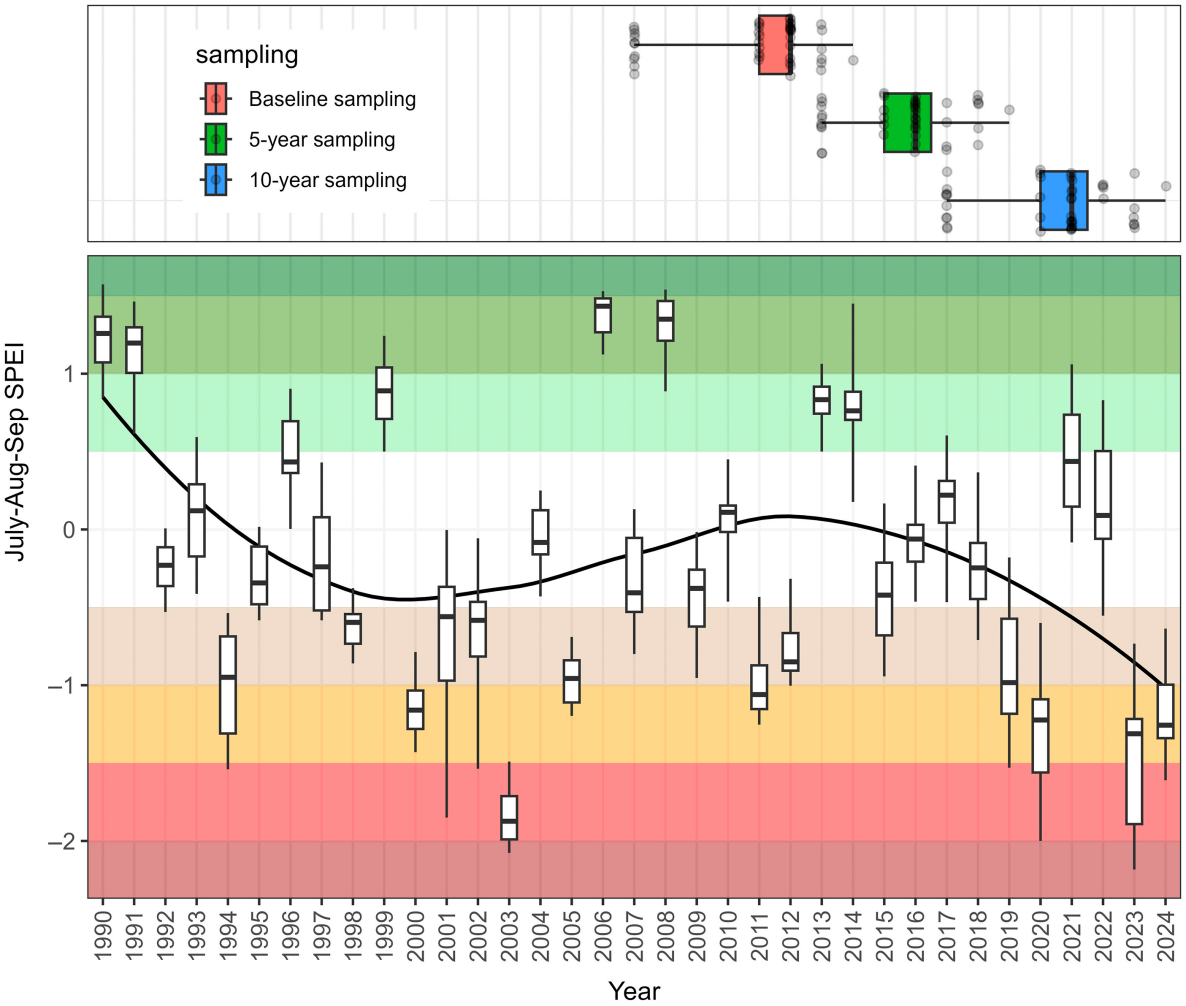
Top panel: Box plots representing years sampled for baseline, 5, and 10‐year measurements of 43 plot pairs in this study. Bottom panel: Box plots of growing season (July–September) standardized precipitation and evapotranspiration index (SPEI) values associated with plot pairs, 1990–2024. Color ramp corresponds to drought categories where −0.5 to 0.5 is normal, positive increments indicate increasing extremity of wetness, and negative increments indicate increasing extremity of dryness.

In the subset of plot pairs (*n* = 10) for which we have 15 years of posttreatment data (Figure 2), declines in encroaching shrub cover due to treatment were sustained across sampling periods (Appendix S1: Table S5). Large increases in both total canopy cover and perennial grass cover were observed in treated plots at 5 years, with little change in control plots. At 10 years, rapid declines in grass cover occurred in treated plots with less severe declines in control plots, but some of the earlier cover gains in treated relative to control plots were sustained at 10 and 15 years. Reference grass cover, in contrast, did not change in treatments at 5 years, while cover in controls was lower. By Year 10, however, treatments and controls did not differ. The overall pattern of declining grass cover over the monitoring period (Figure 2) and weakening of treatment gains (Figure 1) is likely related to increasingly dry conditions during the latter part of the study (Figure 3).

From a multivariate perspective, MRPP indicated that community composition differed between control and treatment plots at 5 years posttreatment. However, differences were not significant at 10 years (Table 2).

**TABLE 2:** Results of multiresponse permutation procedure comparing plant functional groups for controls versus treatments at baseline, 5, and 10 year sample periods.

Sample period	Observed delta		*p*
	Control	Treatment	
Baseline	23.49	22.45	0.597
5‐year	17.49	30.87	0.001
10‐year	26.23	32.41	0.378

*Note*: The observed delta is the weighted mean within‐group distance from cover data for plant functional groups.

**FIGURE 4:**
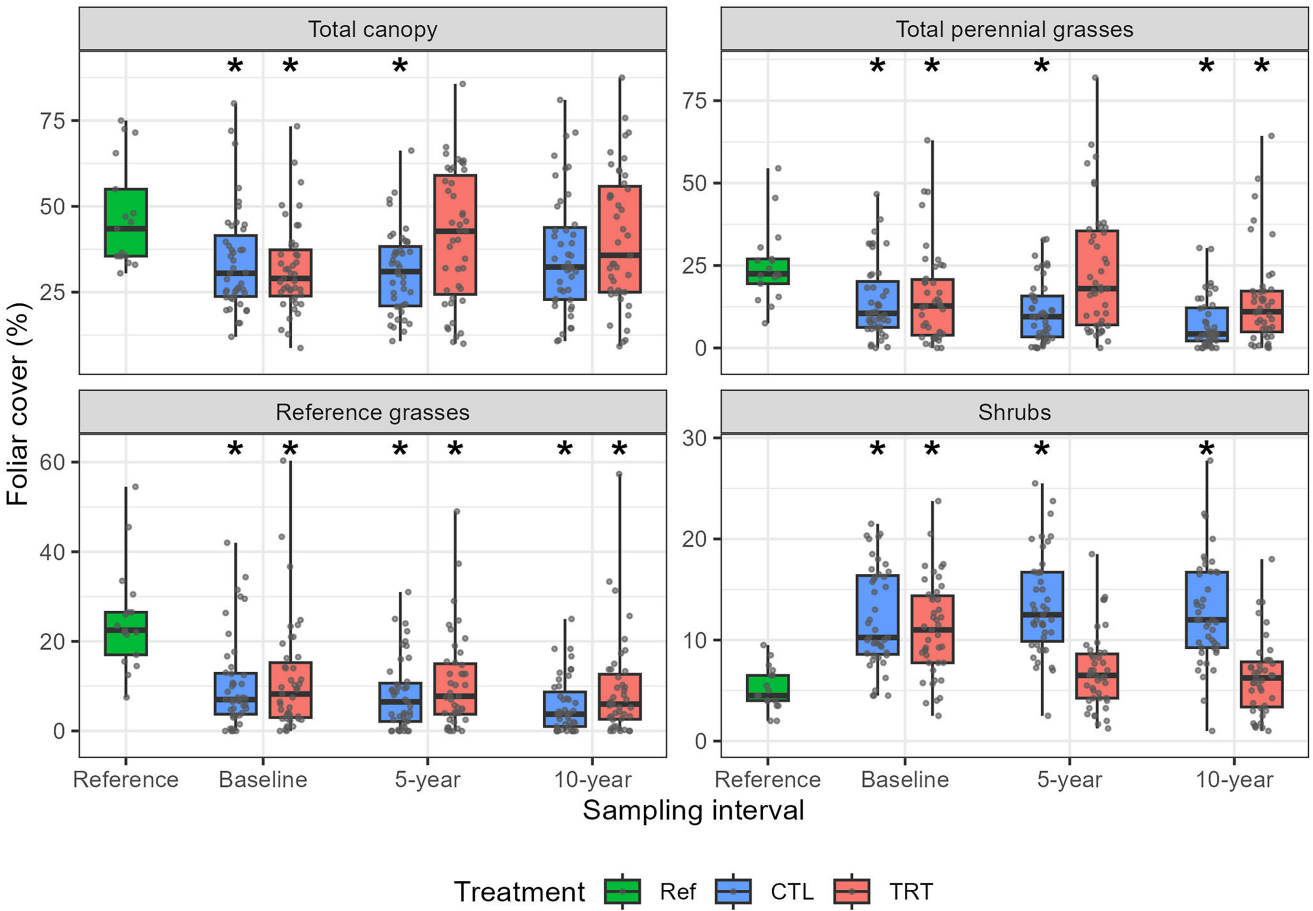
Box plots of cover values for select plant functional groups for reference plots and treated and control plots at baseline, 5‐, and 10‐year sampling periods. Asterisks indicate significant differences (*p* ≤ 0.05) based on Dunnett's tests.

In comparison to our plots representing the reference state, shrub cover was reduced in treatments to levels observed in reference plots (Figure 4). In turn, treated plots attained total canopy cover similar to the reference plots. Perennial grass cover in treated plots was similar to that in reference plots after 5 years, but not at 10 years posttreatment. Reference grass cover in treatments never attained the level observed in reference plots.

**FIGURE 5:**
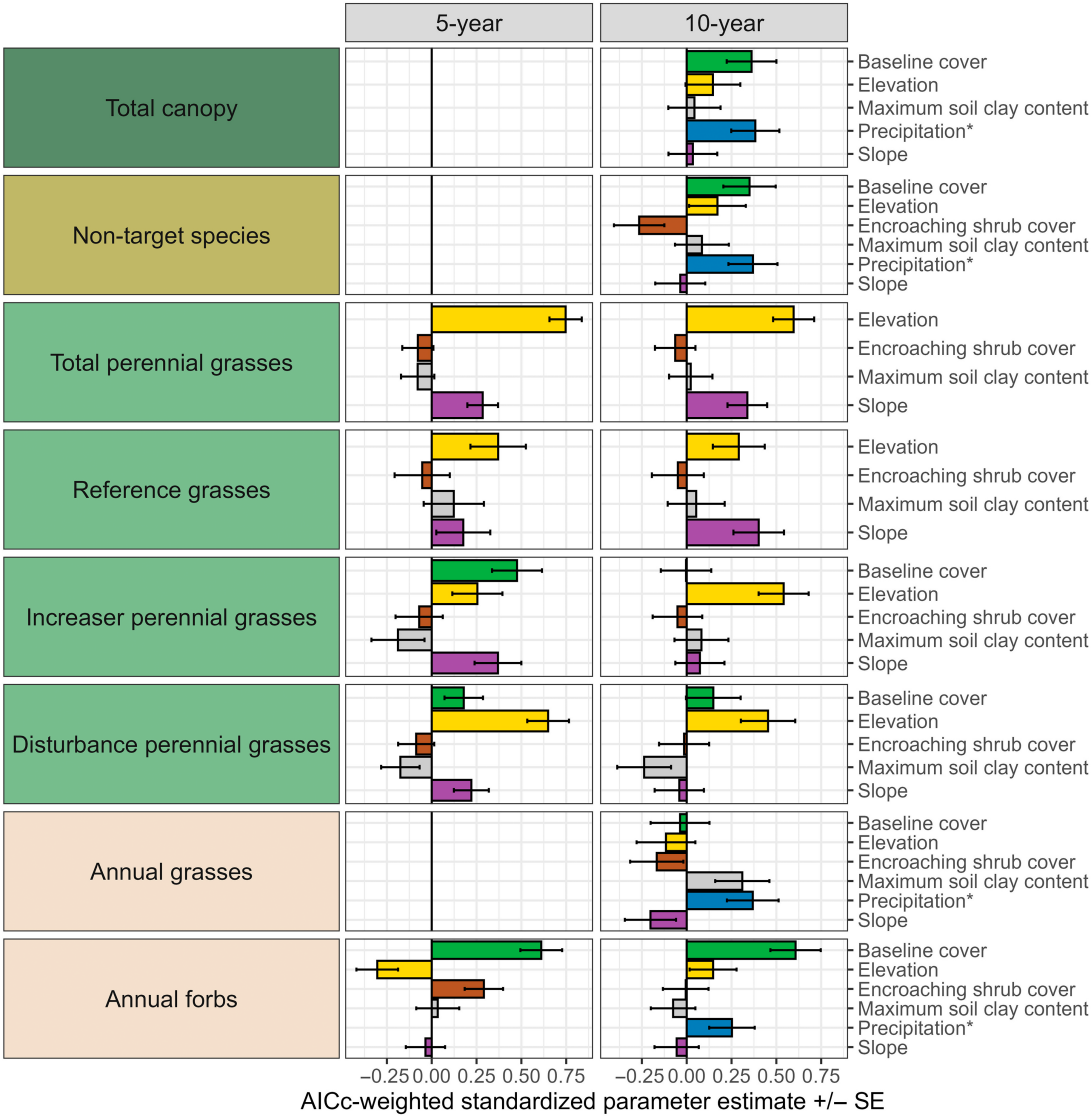
Corrected Akaike information criterion (AICc)‐weighted standardized parameter estimates (mean ± SE) of environmental factors (Appendix S1: Table S2) controlling treatment responses of plant functional groups derived from linear mixed models. *Precipitation refers to one of several possible metrics (Appendix S1: Table S3), where the metric used for a functional group (Appendix S1: Table S4) was determined by the lowest AICc value unless the intercept‐only model was within 2 AICc units of this value. We excluded some environmental factors when they were highly correlated (*r* > 0.40) with other factors, and functional groups were not included if the intercept‐only model was within 2 AICc units of the top model.

The importance of environmental factors driving variation in treatment effects varied among indicators and time periods (Figure 5). For species richness and perennial forbs, the intercept‐only model was best, indicating that none of the measured environmental factors was useful in predicting treatment outcomes. Treatment‐related gains in canopy and nontarget species cover were positively associated with baseline cover and precipitation metrics. Increases in elevation, and sometimes slope, were most strongly associated with gains in total perennial, reference, and disturbance‐adapted grasses. Baseline cover was most important for explaining responses of annual forbs, whereas increasing soil clay content and precipitation explained annual grass gains at 10 years posttreatment. For three indicators (total canopy cover, nontarget species, and annual grasses), significant predictors only emerged after 10 years.

**FIGURE 6:**
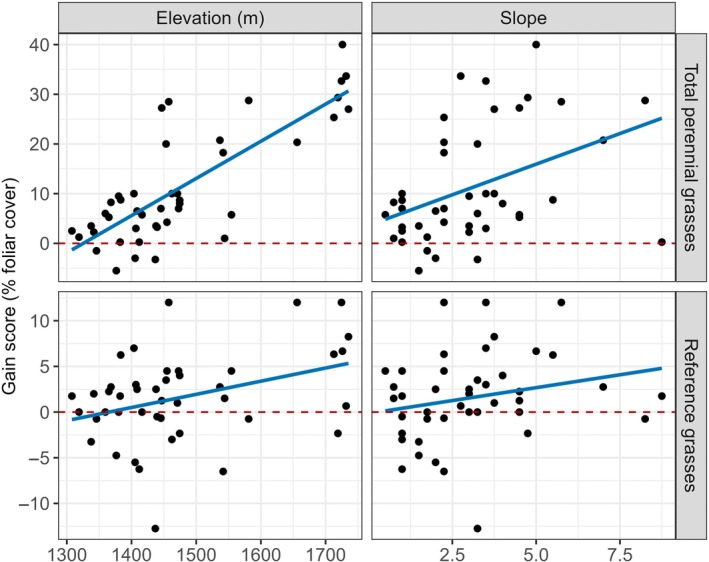
Plots of 5‐year gain scores of select plant functional groups against their most important environmental predictors (see Figure 5).

Plots of treatment‐related gains in total perennial grass and reference grass cover after 5 years (cf. Figure 5, first column) against elevation and slope illustrate an important aspect of variability in treatment effect–environment relationships (Figure 6). While relationships are positive (and for elevation effects on perennial grass gains, strongly so), low elevation and slope values were often associated with negative gain scores, particularly for reference grasses. That is, these treated plots lost cover to a greater degree (or increased cover more slowly) than the paired control plot, indicating that treatments disadvantaged these plant functional groups in those sites.

We additionally tested the effects of grazing utilization classes observed at the time of sampling on treatment‐related gains (Appendix S1: Figure S7). Utilization had no effect on most plant functional groups. Gains in perennial forbs at 5 years, annual forbs at 10 years, and species richness at both 5 and 10 years were greater when utilization was moderate‐heavy compared to none‐light (*p* < 0.05). The opposite pattern was observed for reference grasses at 10 years (*p* = 0.06).

## DISCUSSION

Aside from a few studies (e.g., Ding & Eldridge, 2023), there has been limited evidence about (1) the degree to which removal reverses the impacts of woody plant encroachment, and (2) the conditions under which recovery of pre‐encroachment conditions is optimized. Below, we show how our long‐term, large‐scale experiment provided high‐quality evidence addressing these information gaps.

### Does woody plant removal result in restoration, novel communities, or degradation?

Following our predictions, and consistent with both regional (Buonopane et al., 2005; Chiquoine et al., 2024) and global patterns (Archer et al., 2017; Ding & Eldridge, 2024), perennial grass cover increased with woody plant removal. The plants benefiting most from removal, however, tended to be disturbance‐adapted grasses (at 5 years posttreatment) and annual forbs (Figures 1 and 2). Furthermore, comparisons of the plant communities resulting from woody plant removal with reference sites (Figure 4) indicate that removal did not restore reference grasses to levels expected from our reference sites.

The concern that woody plant removal would inadvertently reduce overall canopy cover, thereby increasing erosion risk (Brock et al., 2014; Ding & Eldridge, 2024; Li et al., 2007), was not supported (Figures 1 and 2). In our larger 10‐year dataset, removal increased total canopy cover soon after treatment, but this increase disappeared at 10 years. In the smaller 15‐year dataset, modest increases in canopy cover due to removal were sustained for 15 years. While we did not record changes in surface roughness and connectivity needed to assess soil erosion potential, the increased canopy cover should reduce, rather than increase, susceptibility to wind erosion as well as susceptibility to raindrop impact and water erosion (Webb et al., 2014). Similarly, the concern that the use of herbicides would inadvertently reduce the cover of valuable forbs (Bennion et al., 2020) was not substantiated. Species richness, annual, and perennial forb cover were either increased or unaffected by treatments, providing no evidence for negative impacts to plant biodiversity and resources used by wildlife.

In contrast to expectations that woody plant removal benefits may last only a few years (Archer et al., 2017), woody plant cover in our study was suppressed with the positive effects of treatments lasting up to 15 years (Figure 2). The success of treatments may be attributable to the effectiveness of tebuthiuron on the target species (Brock et al., 2014), and the episodic nature of recruitment in the target shrub species (Moreno‐de las Heras, 2016), coupled with generally dry conditions (Figure 3). Moreover, shrub recruitment was visually observed in many treatments, so it is unclear how much longer shrub suppression will continue. Longer term persistence of grass gains may require repeated treatment, as observed in other studies (Archer et al., 2011).

Overall, these results suggest that while woody plant removal cannot be considered to restore a reference state in our region due to the continued absence of reference grass species, removal does not—on average—lead to outcomes that local land managers consider to be land degradation (Jones et al., 2024). Furthermore, previous point‐in‐time comparisons of bird communities (Coffman et al., 2014), lizard communities (Cosentino et al., 2013), a keystone rodent species (Cosentino et al., 2014), and ant colonies (McAllister et al., 2014) in treated and untreated areas within the study region indicate benefits of woody plant removal to species of conservation concern and shifts from shrubland to grassland‐associated animal species. The balance between these distinct assemblages can be managed by taking a landscape mosaic approach to woody plant management, involving a strategically designed mix of treated and untreated areas (Archer et al., 2017, Ding & Eldridge, 2024).

Our results align with the global generalization that restoration seldom results in a complete return of biodiversity and ecosystem services observed under reference conditions (Atkinson et al., 2022; Jones et al., 2018; Rey Benayas et al., 2009). They also support the generalization that woody plant removal can result in partial restoration of grassland ecosystem services (Ding & Eldridge, 2023). Our results reinforce the interpretation that woody plant removal in our region produces a novel ecosystem (Coffman et al., 2014). The novel grassland state is less desirable for livestock grazing and biodiversity conservation than the reference grassland once occupying these lands. Nonetheless, the average outcomes of treatments are (for our land managers at least) more desirable than the shrubland state from which they are derived (e.g., Webb et al., 2025), especially in the context of a homogeneous shrub‐dominated landscape (Hobbs et al., 2014; Miller & Bestelmeyer, 2016). Following the Resist‐Accept‐Direct framework (Lynch et al., 2021), woody plant removal can be considered a “Direct” management decision, in which managers “accept that change is inevitable but intervene to steer the transformation toward an ecosystem state with particular structure and function” supporting some, but not all, management objectives.

The mechanisms limiting recovery of the reference state are likely related to complex and incompletely understood ecological thresholds involving a mix of processes. Processes include soil structural changes due to erosion within the shrubland state that prevents grass reestablishment (Bestelmeyer et al., 2006; Herrick et al., 2002), reproductive limitations of key grass species such as when cover becomes too low to sustain adequate recruitment (Svejcar et al., 2015), impacts of wild and managed herbivores on grass recovery (Andreoni et al., 2024), and climate‐driven limitations in water availability to support grass recovery and survival (Gremer et al., 2015).

The potential role of climate in limiting vegetation recovery points to a weakness in our assumptions. Restoration strategies increasingly account for future climatic conditions that are unlike those in the past (Butterfield et al., 2017). Although total canopy cover initially increased due to woody plant removal, the weakening of this increase as aridity increased in the latter part of the study (Figures 1 and 3) suggests that canopy cover might be increasingly negatively affected by treatments if aridity continues to intensify (Seager et al., 2023; Zhuang et al., 2024). In other words, accepting the shrub‐encroached state (Lynch et al., 2021) might prove in the future to be the best of available options to maintain vegetation cover and a relatively lower rate of soil erosion (Fick et al., 2022).

### What conditions maximize successful outcomes of woody plant removal?

The best predictors of treatment‐related gains depended on the plant functional groups in question, but three classes of factors stood out. First, baseline cover was a strong predictor of gains in canopy cover and annual forbs, suggesting that the characteristics of the initial state are useful in site selection for treatments. For example, below a threshold of herbaceous species abundance, recovery may be limited due to feedbacks between patch size and resource availability known to govern dryland plant communities (Kéfi et al., 2024; Svejcar et al., 2015). Second, positive precipitation anomalies increased gains in canopy cover and annual plants. Weather is well known to influence restoration outcomes, but at timescales longer than the seasonal timescales used in seeding decisions (Hagger et al., 2018), forecasts are unreliable. Thus, at best, weather variability can (1) inform grazing management decisions to maximize herbaceous recovery opportunities and (2) retrospectively help explain variations in woody treatment responses in different time periods. Finally, increasing elevation and, to a lesser extent, slope was strongly related to gains in perennial grasses. Higher elevations are associated with wetter and cooler climates and increased soil organic matter that favor grass cover and persistence (Abel et al., 2024; Mata‐González et al., 2002). Slope modulates microclimate and water runoff, potentially favoring grass recruitment in areas with more favorable water balance (Ludwig et al., 2005).

Our metric of posttreatment grazing utilization indicated that heavier grazing was associated with treatment‐related gains in species richness and sometimes forb cover (Appendix S1: Figure S7). There was little evidence for negative effects of moderate‐heavy grazing on plant responses except for a marginal effect on reference grasses at 10 years. Plant species richness and forbs can be favored by grazing‐associated reductions in dominant grasses and increased soil disturbance (Loeser et al., 2007; Török et al., 2024), although only a few forbs in our study were exotic species. It is also possible that causation is reversed—that signs of utilization were more evident in species‐rich plant communities. We further acknowledge that our utilization measurements do not account for most years that grazing occurred, so our assessment of the grazing regime is limited.

Overall, our results suggest that successful woody plant removal outcomes are (1) predictable based on environmental factors, and (2) maximized where (and when) soil moisture is least limiting or the cover of responding plants is highest prior to treatments. The relationships between treatment‐related gains with their most important environmental controls illustrate the utility of data visualization and further analysis in making specific treatment decisions (Figure 6). For example, below 1450 m elevation, responses of perennial and reference grasses were often close to zero or even negative. Note that heavy grazing may, in part, be a cause of negative effects if removal of shrubs makes access to grasses easier for livestock, so attention to posttreatment grazing management is critical (Milchunas & Noy‐Meir, 2002). To operationalize our results for future restoration decisions, managers now use our dataset to define benchmarks and levels of uncertainty for specific plant responses in considering whether or not to treat in a given environmental context (Brudvig & Catano, 2024).

## IMPLICATIONS FOR MANAGEMENT

Our management experiment showed that woody plant removal, at least over 10–15‐year timescales, may not be able to recover reference states due either to the development of ecological thresholds during encroachment (Suding & Hobbs, 2009) or because the climatic context necessary for recovery of historical states has changed (Harris et al., 2006). The question then becomes whether woody plant removal creates other sustainable benefits for stakeholders, recognizing that (1) stakeholders vary in the benefits expected and cost–benefit evaluations (Fox & Cundill, 2018), and (2) the ability to achieve those benefits varies over time and space. We found that novel ecosystems and landscape perspectives (Hobbs et al., 2014) alongside the Resist‐Accept‐Direct framework (Lynch et al., 2021) provide a useful way to frame these questions.

To help land managers navigate restoration decisions in dynamic ecosystems, our study demonstrates how long‐term, large‐scale monitoring experiments embedded in operational restoration actions can resolve key uncertainties about the overall efficacy of a restoration program as well as the environmental conditions in which individual actions are most successful (Brudvig et al., 2021). Several factors were critical to the success of our research. First, scientists and land managers maintained a long‐term collaboration, despite personnel turnover, to ensure that on‐the‐ground treatments planned by managers across a vast area were part of the experiment. Frequent communication and partnership with boundary spanning individuals made this possible over 15 years (Bestelmeyer et al., 2019). Second, standardized “core” monitoring methods were essential to ensure scalability, quality, and comparability of measurements, including reference sites that were sampled before this study (McCord et al., 2023). Expanding standardized vegetation monitoring data that have been gathered at over 100,000 sites globally (most in the United States) and integrated in a single database constitute a powerful tool for comparing restoration outcomes over time (Gann et al., 2019). Third, rather than choosing indicators of interest exclusively to the scientific community, we focused on indicators of interest to stakeholders that were easy to measure and interpret and therefore would be most likely to influence future decisions (Reed et al., 2008). While our indicators were not exhaustive, they have proved sufficient for decision‐making when coupled with the results of shorter term studies of biodiversity indicators that are more costly to monitor (Cosentino et al., 2014). Attention to these three principles will strengthen the collaborative adaptive management and effectiveness of restoration investments.

## AUTHOR CONTRIBUTIONS

Brandon T. Bestelmeyer: conceptualization, funding acquisition, project administration, writing—original draft preparation. Laura M. Burkett: data curation, investigation, project administration, writing—original draft preparation. Darren James: data curation, formal analysis, visualization, writing—original draft preparation. Juan Gamon: funding acquisition, resources. Robert L. Schooley: validation, writing—review and editing.

## CONFLICT OF INTEREST STATEMENT

The authors declare no conflicts of interest.

## Supporting information


Appendix S1.

Appendix S2.

## Data Availability

Data (Bestelmeyer et al., 2025) are available in the Environmental Data Initiative's EDI Data Portal at https://doi.org/10.6073/pasta/c262ad66f6acdea2fee3774713b1e3a4. Monthly precipitation rasters were obtained using the PRISM R package (Hart & Bell, 2015).

## References

[eap70240-bib-0001] Abatzoglou, J. T. , D. J. McEvoy , and K. T. Redmond . 2017. “The West Wide Drought Tracker: Drought Monitoring at Fine Spatial Scales.” Bulletin of the American Meteorological Society 98: 1815–1820.

[eap70240-bib-0002] Abel, C. , F. T. Maestre , M. Berdugo , T. Tagesson , A. M. Abdi , S. Horion , and R. Fensholt . 2024. “Vegetation Resistance to Increasing Aridity when Crossing Thresholds Depends on Local Environmental Conditions in Global Drylands.” Communications Earth & Environment 5: 379.

[eap70240-bib-0003] Anadón, J. D. , O. E. Sala , B. L. Turner , and E. M. Bennett . 2014. “Effect of Woody‐Plant Encroachment on Livestock Production in North and South America.” Proceedings of the National Academy of Sciences 111: 12948–12953. 10.1073/pnas.1320585111 PMC4156688 25136084

[eap70240-bib-0004] Andreoni, K. J. , B. T. Bestelmeyer , D. C. Lightfoot , and R. L. Schooley . 2024. “Effects of Multiple Mammalian Herbivores and Climate on Grassland–Shrubland Transitions in the Chihuahuan Desert.” Ecology 105: e4460. 39470114 10.1002/ecy.4460 PMC11610684

[eap70240-bib-0005] Aoki, L. R. , K. J. McGlathery , P. L. Wiberg , and A. Al‐Haj . 2020. “Depth Affects Seagrass Restoration Success and Resilience to Marine Heat Wave Disturbance.” Estuaries and Coasts 43: 316–328.

[eap70240-bib-0006] Archer, S. A. , K. W. Davies , T. E. Fulbright , K. C. McDaniel , B. P. Wilcox , and K. I. Predick . 2011. “Brush Management as a Rangeland Conservation Tool: A Critical Evaluation.” In Conservation Benefits of Rangeland Practices: Assessment, Recommendations, and Knowledge Gaps, edited by D. D. Briske , 105–170. Lawrence, KS: Allen Press.

[eap70240-bib-0007] Archer, S. R. , E. M. Andersen , K. I. Predick , S. Schwinning , R. J. Steidl , and S. R. Woods . 2017. “Woody Plant Encroachment: Causes and Consequences.” In Rangeland Systems: Processes, Management and Challenges, edited by D. D. Briske , 25–84. Cham: Springer International Publishing.

[eap70240-bib-0008] Archer, S. R. , D. P. C. Peters , N. D. Burruss , and J. Yao . 2022. “Mechanisms and Drivers of Alternative Shrubland States.” Ecosphere 13: e3987.

[eap70240-bib-0009] Archer, S. R. , and K. I. Predick . 2014. “An Ecosystem Services Perspective on Brush Management: Research Priorities for Competing Land‐Use Objectives.” Journal of Ecology 102: 1394–1407.

[eap70240-bib-0010] Atkinson, J. , L. A. Brudvig , M. Mallen‐Cooper , S. Nakagawa , A. T. Moles , and S. P. Bonser . 2022. “Terrestrial Ecosystem Restoration Increases Biodiversity and Reduces its Variability, but Not to Reference Levels: A Global Meta‐Analysis.” Ecology Letters 25: 1735–1737. 10.1111/ele.14025 PMC9320827 35559594

[eap70240-bib-0011] Bennion, L. D. , J. A. Ferguson , L. F. New , and C. B. Schultz . 2020. “Community‐Level Effects of Herbicide‐Based Restoration Treatments: Structural Benefits but at What Cost?” Restoration Ecology 28: 553–563.

[eap70240-bib-0012] Bestelmeyer, B. , L. M. Burkett , and D. James . 2025. “Large‐Scale Woody Plant Removal Outcomes in Southern New Mexico, USA, 2007‐2024, Version 1.” Environmental Data Initiative. 10.6073/pasta/c262ad66f6acdea2fee3774713b1e3a4.

[eap70240-bib-0013] Bestelmeyer, B. T. , L. M. Burkett , L. Lister , J. R. Brown , and R. L. Schooley . 2019. “Collaborative Approaches to Strengthen the Role of Science in Rangeland Conservation.” Rangelands 41: 218–226.

[eap70240-bib-0014] Bestelmeyer, B. T. , M. C. Duniway , D. K. James , L. M. Burkett , and K. M. Havstad . 2013. “A Test of Critical Thresholds and their Indicators in a Desertification‐Prone Ecosystem: More Resilience than we Thought.” Ecology Letters 16: 339–345. 23216915 10.1111/ele.12045

[eap70240-bib-0015] Bestelmeyer, B. T. , D. P. C. Peters , S. R. Archer , D. M. Browning , G. S. Okin , R. L. Schooley , and N. P. Webb . 2018. “The Grassland‐Shrubland Regime Shift in the Southwestern United States: Misconceptions and their Implications for Management.” Bioscience 68: 678–690.

[eap70240-bib-0016] Bestelmeyer, B. T. , J. P. Ward , J. E. Herrick , and A. J. Tugel . 2006. “Fragmentation Effects on Soil Aggregate Stability in a Patchy Arid Grassland.” Rangeland Ecology & Management 59: 406–415.

[eap70240-bib-0017] Block, W. M. , A. B. Franklin , J. P. Ward, Jr. , J. L. Ganey , and G. C. White . 2001. “Design and Implementation of Monitoring Studies to Evaluate the Success of Ecological Restoration on Wildlife.” Restoration Ecology 9: 293–303.

[eap70240-bib-0018] Briske, D. D. , B. T. Bestelmeyer , J. R. Brown , M. W. Brunson , T. L. Thurow , and J. A. Tanaka . 2017. “Assessment of USDA‐NRCS Rangeland Conservation Programs: Recommendation for an Evidence‐Based Conservation Platform.” Ecological Applications 27: 94–104. 27870290 10.1002/eap.1414

[eap70240-bib-0019] Brock, J. , B. Brandau , D. Arthun , A. L. Humphrey , G. Dominguez , and A. Jacobs . 2014. “Long‐Term Results of Tebuthiuron Herbicide Treatment on Creosote Bush (*Larrea tridentata*) in Southeast Arizona, USA.” Journal of Arid Environments 110: 44–46.

[eap70240-bib-0020] Brudvig, L. A. , R. S. Barak , J. T. Bauer , T. T. Caughlin , D. C. Laughlin , L. Larios , J. W. Matthews , K. L. Stuble , N. E. Turley , and C. R. Zirbel . 2017. “Interpreting Variation to Advance Predictive Restoration Science.” Journal of Applied Ecology 54: 1018–1027.

[eap70240-bib-0021] Brudvig, L. A. , and C. P. Catano . 2024. “Prediction and Uncertainty in Restoration Science.” Restoration Ecology 32: e13380.

[eap70240-bib-0022] Brudvig, L. A. , N. E. Turley , S. L. Bartel , L. Bell‐Dereske , S. Breland , E. I. Damschen , S. E. Evans , et al. 2021. “Large Ecosystem‐Scale Effects of Restoration Fail to Mitigate Impacts of Land‐Use Legacies in Longleaf Pine Savannas.” Proceedings of the National Academy of Sciences 118: e2020935118. 10.1073/pnas.2020935118 PMC8092381 33875596

[eap70240-bib-0023] Buffington, L. C. , and C. H. Herbel . 1965. “Vegetational Changes on a Semidesert Grassland Range from 1858 to 1963.” Ecological Monographs 35: 139–164.

[eap70240-bib-0024] Buonopane, M. , L. F. Huenneke , and M. Remmenga . 2005. “Community Response to Removals of Plant Functional Groups and Species from a Chihuahuan Desert Shrubland.” Oikos 110: 67–80.

[eap70240-bib-0025] Butterfield, B. J. , S. M. Copeland , S. M. Munson , C. M. Roybal , and T. E. Wood . 2017. “Prestoration: Using Species in Restoration that Will Persist Now and into the Future.” Restoration Ecology 25: S155–S163.

[eap70240-bib-0026] Chiquoine, L. P. , S. R. Abella , C. D. Schelz , M. F. Medrano , and N. A. Fisichelli . 2024. “Restoring Historical Grasslands in a Desert National Park: Resilience or Unrecoverable States in an Emerging Climate?” Biological Conservation 289: 110387.

[eap70240-bib-0027] Christensen, E. M. , D. K. James , R. M. Randall , and B. T. Bestelmeyer . 2023. “Abrupt Transitions in a Southwest USA Desert Grassland Related to the Pacific Decadal Oscillation.” Ecology 104: e4065. 37186307 10.1002/ecy.4065

[eap70240-bib-0028] Coffman, J. M. , B. T. Bestelmeyer , J. F. Kelly , T. F. Wright , and R. L. Schooley . 2014. “Restoration Practices Have Positive Effects on Breeding Bird Species of Concern in the Chihuahuan Desert.” Restoration Ecology 22: 336–344. 32327918 10.1111/rec.12081 PMC7169160

[eap70240-bib-0029] Cooke, S. J. , J. R. Bennett , and H. P. Jones . 2019. “We Have a Long Way to Go if we Want to Realize the Promise of the “Decade on Ecosystem Restoration”.” Conservation Science and Practice 1: e129.

[eap70240-bib-0030] Cosentino, B. , R. Schooley , B. Bestelmeyer , J. Kelly , and J. Coffman . 2014. “Constraints and Time Lags for Recovery of a Keystone Species (*Dipodomys spectabilis*) after Landscape Restoration.” Landscape Ecology 29: 665–675.

[eap70240-bib-0031] Cosentino, B. J. , R. L. Schooley , B. T. Bestelmeyer , and J. M. Coffman . 2013. “Response of Lizard Community Structure to Desert Grassland Restoration Mediated by a Keystone Rodent.” Biodiversity and Conservation 22: 921–935.

[eap70240-bib-0032] Ding, J. , and D. Eldridge . 2023. “The Success of Woody Plant Removal Depends on Encroachment Stage and Plant Traits.” Nature Plants 9: 58–67. 36543937 10.1038/s41477-022-01307-7

[eap70240-bib-0033] Ding, J. , and D. J. Eldridge . 2019. “Contrasting Global Effects of Woody Plant Removal on Ecosystem Structure, Function and Composition.” Perspectives in Plant Ecology, Evolution and Systematics 39: 125460.

[eap70240-bib-0034] Ding, J. , and D. J. Eldridge . 2024. “Woody Encroachment: Social–Ecological Impacts and Sustainable Management.” Biological Reviews 99: 1909–1926. 38961449 10.1111/brv.13104

[eap70240-bib-0035] Ding, J. , S. K. Travers , M. Delgado‐Baquerizo , and D. J. Eldridge . 2020. “Multiple Trade‐Offs Regulate the Effects of Woody Plant Removal on Biodiversity and Ecosystem Functions in Global Rangelands.” Global Change Biology 26: 709–720. 31518466 10.1111/gcb.14839

[eap70240-bib-0036] Eldridge, D. J. , M. A. Bowker , F. T. Maestre , E. Roger , J. F. Reynolds , and W. G. Whitford . 2011. “Impacts of Shrub Encroachment on Ecosystem Structure and Functioning: Towards a Global Synthesis.” Ecology Letters 14: 709–722. 21592276 10.1111/j.1461-0248.2011.01630.x PMC3563963

[eap70240-bib-0037] Eldridge, D. J. , and J. Ding . 2021. “Remove or Retain: Ecosystem Effects of Woody Encroachment and Removal Are Linked to Plant Structural and Functional Traits.” New Phytologist 229: 2637–2646. 33118178 10.1111/nph.17045

[eap70240-bib-0038] Ellison, A. M. 2019. “Foundation Species, Non‐trophic Interactions, and the Value of Being Common.” iScience 13: 254–268. 30870783 10.1016/j.isci.2019.02.020 PMC6416672

[eap70240-bib-0039] Fick, S. E. , T. W. Nauman , C. C. Brungard , and M. C. Duniway . 2022. “What Determines the Effectiveness of Pinyon‐Juniper Clearing Treatments? Evidence from the Remote Sensing Archive and Counter‐Factual Scenarios.” Forest Ecology and Management 505: 119879.

[eap70240-bib-0040] Fox, H. , and G. Cundill . 2018. “Towards Increased Community‐Engaged Ecological Restoration: A Review of Current Practice and Future Directions.” Ecological Restoration 36: 208–218.

[eap70240-bib-0041] Gann, G. D. , T. McDonald , B. Walder , J. Aronson , C. R. Nelson , J. Jonson , J. G. Hallett , et al. 2019. “International Principles and Standards for the Practice of Ecological Restoration.” Restoration Ecology 27: S1–S46.

[eap70240-bib-0042] Giardina, C. P. , C. M. Litton , J. M. Thaxton , S. Cordell , L. J. Hadway , and D. R. Sandquist . 2007. “Science Driven Restoration: A Candle in a Demon Haunted World ‐ Response to Cabin (2007).” Restoration Ecology 15: 171–176.

[eap70240-bib-0043] Gremer, J. R. , J. B. Bradford , S. M. Munson , and M. C. Duniway . 2015. “Desert Grassland Responses to Climate and Soil Moisture Suggest Divergent Vulnerabilities across the Southwestern United States.” Global Change Biology 21: 4049–4062. 26183431 10.1111/gcb.13043

[eap70240-bib-0044] Hagger, V. , J. Dwyer , L. Shoo , and K. Wilson . 2018. “Use of Seasonal Forecasting to Manage Weather Risk in Ecological Restoration.” Ecological Applications 28: 1797–1807. 30024642 10.1002/eap.1769

[eap70240-bib-0045] Harris, J. A. , R. J. Hobbs , E. Higgs , and J. Aronson . 2006. “Ecological Restoration and Global Climate Change.” Restoration Ecology 14: 170–176.

[eap70240-bib-0046] Hart, E. M. , and K. Bell . 2015. “prism: Download Data from the Oregon Prism Project.” R Package Version 0.0.6. 10.5281/zenodo.33663.

[eap70240-bib-0047] Herrick, J. E. , J. R. Brown , A. J. Tugel , P. L. Shaver , and K. M. Havstad . 2002. “Application of Soil Quality to Monitoring and Management.” Agronomy Journal 94: 3–11.

[eap70240-bib-0048] Herrick, J. E. , K. M. Havstad , and A. Rango . 2006. “Remediation Research in the Jornada Basin: Past and Future.” In Structure and Function of a Chihuahuan Desert Ecosystem: The Jornada Basin LTER, edited by K. M. Havstad , W. H. Schlesinger , and L. F. Huenneke , 278–304. New York, NY: Oxford University Press.

[eap70240-bib-0049] Herrick, J. E. , J. W. V. Zee , S. E. McCord , E. M. Courtright , J. W. Karl , and L. M. Burkett . 2017. “Monitoring Manual for Grassland, Shrubland and Savanna Ecosystems.” In Core Methods, 2nd ed., Vol. I. Las Cruces, NM: USDA‐ARS Jornada Experimental Range.

[eap70240-bib-0050] Hobbs, R. J. , E. Higgs , C. M. Hall , P. Bridgewater , F. S. Chapin, III , E. C. Ellis , J. J. Ewel , et al. 2014. “Managing the Whole Landscape: Historical, Hybrid, and Novel Ecosystems.” Frontiers in Ecology and the Environment 12: 557–564.

[eap70240-bib-0051] Holechek, J. L. , and D. Galt . 2000. “Grazing intensity guidelines.” Rangelands 22: 11–14.

[eap70240-bib-0052] Jacobs, B. F. , W. H. Romme , and C. D. Allen . 2008. “Mapping “Old” Vs. “Young” piñon–Juniper Stands with a Predictive Topo‐Climatic Model.” Ecological Applications 18: 1627–1641. 18839759 10.1890/07-0847.1

[eap70240-bib-0053] Jones, H. P. , P. C. Jones , E. B. Barbier , R. C. Blackburn , J. M. Rey Benayas , K. D. Holl , M. McCrackin , P. Meli , D. Montoya , and D. M. Mateos . 2018. “Restoration and Repair of Earth's Damaged Ecosystems.” Proceedings of the Royal Society B: Biological Sciences 285: 20172577. 10.1098/rspb.2017.2577 PMC5832705 29491171

[eap70240-bib-0054] Jones, S. A. , L. A. Fisher , J. R. Soto , and S. R. Archer . 2024. “Shrub Encroachment and Stakeholder Perceptions of Rangeland Ecosystem Services: Balancing Conservation and Management?” Ecology and Society 29: 3.

[eap70240-bib-0055] Kéfi, S. , A. Génin , A. Garcia‐Mayor , E. Guirado , J. S. Cabral , M. Berdugo , J. Guerber , R. Solé , and F. T. Maestre . 2024. “Self‐Organization as a Mechanism of Resilience in Dryland Ecosystems.” Proceedings of the National Academy of Sciences 121: e2305153121. 10.1073/pnas.2305153121 PMC10861902 38300860

[eap70240-bib-0056] Krzywicka, A. E. , G. E. Pociask , D. A. Grimley , and J. W. Matthews . 2017. “Hydrology and Soil Magnetic Susceptibility as Predictors of Planted Tree Survival in a Restored Floodplain Forest.” Ecological Engineering 103: 275–287.

[eap70240-bib-0057] Lenth, R. 2025. “emmeans: Estimated Marginal Means, Aka Least‐Squares Means.” R Package Version 2.3.3.

[eap70240-bib-0058] Li, J. , G. S. Okin , L. Alvarez , and H. Epstein . 2007. “Quantitative Effects of Vegetation Cover on Wind Erosion and Soil Nutrient Loss in a Desert Grassland of Southern New Mexico, USA.” Biogeochemistry 85: 317–332.

[eap70240-bib-0059] Loeser, M. R. R. , T. D. Sisk , and T. E. Crews . 2007. “Impact of Grazing Intensity during Drought in an Arizona Grassland.” Conservation Biology 21: 87–97. 17298514 10.1111/j.1523-1739.2006.00606.x

[eap70240-bib-0060] Ludwig, J. A. , B. P. Wilcox , D. D. Breshears , D. J. Tongway , and A. C. Imeson . 2005. “Vegetation Patches and Runoff‐Erosion as Interacting Ecohydrological Processes in Semiarid Landscapes.” Ecology 86: 288–297.

[eap70240-bib-0061] Lynch, A. J. , L. M. Thompson , E. A. Beever , D. N. Cole , A. C. Engman , C. Hawkins Hoffman , S. T. Jackson , et al. 2021. “Managing for RADical Ecosystem Change: Applying the Resist‐Accept‐Direct (RAD) Framework.” Frontiers in Ecology and the Environment 19: 461–469.

[eap70240-bib-0062] Macías‐Duarte, A. , A. O. Panjabi , D. B. Pool , I. Ruvalcaba‐Ortega , and G. J. Levandoski . 2018. “Fall Vegetative Cover and Summer Precipitation Predict Abundance of Wintering Grassland Birds across the Chihuahuan Desert.” Journal of Arid Environments 156: 41–49.

[eap70240-bib-0063] Maestre, F. T. , D. J. Eldridge , S. Soliveres , S. Kéfi , M. Delgado‐Baquerizo , M. A. Bowker , P. García‐Palacios , et al. 2016. “Structure and Functioning of Dryland Ecosystems in a Changing World.” Annual Review of Ecology, Evolution, and Systematics 47: 215–237. 10.1146/annurev-ecolsys-121415-032311 PMC5321561 28239303

[eap70240-bib-0064] Manning, P. , F. van der Plas , S. Soliveres , E. Allan , F. T. Maestre , G. Mace , M. J. Whittingham , and M. Fischer . 2018. “Redefining Ecosystem Multifunctionality.” Nature Ecology & Evolution 2: 427–436. 29453352 10.1038/s41559-017-0461-7

[eap70240-bib-0065] Mata‐González, R. , B. Figueroa‐Sandoval , F. Clemente , and M. Manzano . 2007. “Vegetation Changes after Livestock Grazing Exclusion and Shrub Control in the Southern Chihuahuan Desert.” Western North American Naturalist 67: 63–70.

[eap70240-bib-0066] Mata‐González, R. , R. D. Pieper , and M. M. Cárdenas . 2002. “Vegetation Patterns as Affected by Aspect and Elevation in Small Desert Mountains.” The Southwestern Naturalist 47: 440–448.

[eap70240-bib-0067] Mazerolle, M. 2023. “AICcmodavg: Model Selection and Multimodel Inference Based on (Q)AIC(c).” R Package Version 2.3.3.

[eap70240-bib-0068] McAllister, M. M. , R. L. Schooley , B. T. Bestelmeyer , J. M. Coffman , and B. J. Cosentino . 2014. “Effects of Grassland Restoration Efforts on Mound‐Building Ants in the Chihuahuan Desert.” Journal of Arid Environments 111: 79–83.

[eap70240-bib-0069] McCord, S. E. , N. P. Webb , B. T. Bestelmeyer , K. Bonefont , J. R. Brehm , J. Brown , E. M. Courtright , et al. 2023. “The Landscape Data Commons: A System for Standardizing, Accessing, and Applying Large Environmental Datasets for Agroecosystem Research and Management.” Agricultural & Environmental Letters 8: e20120.

[eap70240-bib-0070] Milchunas, D. G. , and I. Noy‐Meir . 2002. “Grazing Refuges, External Avoidance of Herbivory and Plant Diversity.” Oikos 99: 113–130.

[eap70240-bib-0071] Miller, J. R. , and B. T. Bestelmeyer . 2016. “What's Wrong with Novel Ecosystems, Really?” Restoration Ecology 24: 577–582.

[eap70240-bib-0072] Moreno‐de las Heras, M. , L. Turnbull , and J. Wainwright . 2016. “Seed‐Bank Structure and Plant‐Recruitment Conditions Regulate the Dynamics of a Grassland‐Shrubland Chihuahuan Ecotone.” Ecology 97: 2303–2318. 27859083 10.1002/ecy.1446

[eap70240-bib-0073] Munson, S. M. , E. O. Yackulic , L. S. Bair , S. M. Copeland , and K. L. Gunnell . 2020. “The Biggest Bang for the Buck: Cost‐Effective Vegetation Treatment Outcomes across Drylands of the Western United States.” Ecological Applications 30: e02151. 32342581 10.1002/eap.2151

[eap70240-bib-0074] Norström, A. V. , C. Cvitanovic , M. F. Löf , S. West , C. Wyborn , P. Balvanera , A. T. Bednarek , et al. 2020. “Principles for Knowledge co‐Production in Sustainability Research.” Nature Sustainability 3: 182–190.

[eap70240-bib-0075] Ogle, K. , and J. F. Reynolds . 2004. “Plant Responses to Precipitation in Desert Ecosystems: Integrating Functional Types, Pulses, Thresholds, and Delays.” Oecologia 141: 282–294. 15007725 10.1007/s00442-004-1507-5

[eap70240-bib-0076] Oksanen, J. , G. Simpson , F. Blanchet , R. Kindt , P. Legendre , P. Minchin , R. O'Hara , et al. 2025. “vegan: Community Ecology Package.” R package version 2.6‐10.

[eap70240-bib-0077] Parr, C. L. , M. te Beest , and N. Stevens . 2024. “Conflation of Reforestation with Restoration Is Widespread.” Science 383: 698–701. 38359128 10.1126/science.adj0899

[eap70240-bib-0078] Perring, M. P. , J. Jonson , D. Freudenberger , R. Campbell , M. Rooney , R. J. Hobbs , and R. J. Standish . 2015. “Soil‐Vegetation Type, Stem Density and Species Richness Influence Biomass of Restored Woodland in South‐Western Australia.” Forest Ecology and Management 344: 53–62.

[eap70240-bib-0079] Pierce, N. A. , S. R. Archer , B. T. Bestelmeyer , and D. K. James . 2019. “Grass‐Shrub Competition in Arid Lands: An Overlooked Driver in Grassland–Shrubland State Transition?” Ecosystems 22: 619–628.

[eap70240-bib-0080] PRISM Climate Group . 2025. PRISM Gridded Climate Data. Corvallis, OR: Oregon State University.

[eap70240-bib-0081] R Core Team . 2024. R: A Language and Environment for Statistical Computing. Vienna, Austria: R Foundation for Statistical Computing.

[eap70240-bib-0082] Reed, M. S. , A. J. Dougill , and T. R. Baker . 2008. “Participatory Indicator Development: What Can Ecologists and Local Communities Learn from each Other?” Ecological Applications 18: 1253–1269. 18686585 10.1890/07-0519.1

[eap70240-bib-0083] Rey Benayas, J. M. , A. C. Newton , A. Diaz , and J. M. Bullock . 2009. “Enhancement of Biodiversity and Ecosystem Services by Ecological Restoration: A Meta‐Analysis.” Science 325: 1121–1124. 19644076 10.1126/science.1172460

[eap70240-bib-0084] Roccaforte, J. P. , D. W. Huffman , K. C. Rodman , J. E. Crouse , R. J. Pedersen , D. P. Normandin , and P. Z. Fulé . 2024. “Long‐Term Ecological Responses to Landscape‐Scale Restoration in a Western United States Dry Forest.” Restoration Ecology 32: e14181.

[eap70240-bib-0085] Romig, K. B. , D. K. James , C. J. Maxwell , B. T. Bestelmeyer , J. R. Brown , S. W. Salley , and A. M. Faist . 2025. “Hidden Biodiversity: Dryland Soil Seed Banks across Ecological Sites and States.” Journal of Arid Environments 227: 105307.

[eap70240-bib-0086] Schoeneberger, P. J. , D. A. Wysocki , and E. C. Benham . 2012. Field Book for Describing and Sampling Soils. Washington, DC: Government Printing Office.

[eap70240-bib-0087] Seager, R. , M. Ting , P. Alexander , H. Liu , J. Nakamura , C. Li , and M. Newman . 2023. “Ocean‐Forcing of Cool Season Precipitation Drives Ongoing and Future Decadal Drought in Southwestern North America.” npj Climate and Atmospheric Science 6: 141.

[eap70240-bib-0088] Shackelford, N. , J. Dudney , M. M. Stueber , V. M. Temperton , and K. L. Suding . 2024. “Measuring at all Scales: Sourcing Data for more Flexible Restoration References.” Restoration Ecology 32: e13541.

[eap70240-bib-0089] Steele, C. M. , B. T. Bestelmeyer , L. M. Burkett , P. L. Smith , and S. Yanoff . 2012. “Spatially Explicit Representation of State‐and‐Transition Models.” Rangeland Ecology & Management 65: 213–222.

[eap70240-bib-0090] Suding, K. N. , and R. J. Hobbs . 2009. “Threshold Models in Restoration and Conservation: A Developing Framework.” Trends in Ecology & Evolution 24: 271–279. 19269057 10.1016/j.tree.2008.11.012

[eap70240-bib-0091] Svejcar, L. , B. Bestelmeyer , M. Duniway , and D. James . 2015. “Scale‐Dependent Feedbacks between Patch Size and Plant Reproduction in Desert Grassland.” Ecosystems 18: 146–153.

[eap70240-bib-0092] Török, P. , R. Lindborg , D. Eldridge , and R. Pakeman . 2024. “Grazing Effects on Vegetation: Biodiversity, Management, and Restoration.” Applied Vegetation Science 27: e12794.

[eap70240-bib-0093] USDA Natural Resources Conservation Service . 2021. “RCA Data Viewer.” https://www.nrcs.usda.gov/resources/data-and-reports/rca-data-viewer.

[eap70240-bib-0094] Webb, N. P. , J. E. Herrick , and M. C. Duniway . 2014. “Ecological Site‐Based Assessments of Wind and Water Erosion: Informing Accelerated Soil Erosion Management in Rangelands.” Ecological Applications 24: 1405–1420. 29160663 10.1890/13-1175.1

[eap70240-bib-0095] Webb, N. P. , B. Wheeler , B. L. Edwards , J. W. Schallner , N. Macanowicz , J. W. Van Zee , E. M. Courtright , et al. 2025. “Magnitude Shifts in Aeolian Sediment Transport Associated with Degradation and Restoration Thresholds in Drylands.” Journal of Geophysical Research: Biogeosciences 130: e2024JG008581.

[eap70240-bib-0096] Wickham, H. , M. Averick , J. Bryan , W. Chang , L. McGowan , R. François , G. Grolemund , et al. 2019. “Welcome to the Tidyverse.” Journal of Open Source Software 4: 1686.

[eap70240-bib-0097] Yao, J. , D. C. Peters , K. Havstad , R. Gibbens , and J. Herrick . 2006. “Multi‐Scale Factors and Long‐Term Responses of Chihuahuan Desert Grasses to Drought.” Landscape Ecology 21: 1217–1231.

[eap70240-bib-0098] Zhuang, Y. , R. Fu , J. Lisonbee , A. M. Sheffield , B. A. Parker , and G. Deheza . 2024. “Anthropogenic Warming Has Ushered in an Era of Temperature‐Dominated Droughts in the Western United States.” Science Advances 10: eadn9389. 39504363 10.1126/sciadv.adn9389 PMC11540010

