# Supplementary material for: Large‐scale experimental evaluation of woody plant removal in desert grassland: Restoration, novelty, or degradation?
Source: Ecol Appl. 2026 Apr 21;36:e70240. doi: 10.1002/eap.70240 (PMC13099281; doi:10.1002/eap.70240)
Supplement: Supplementary file 1 — Appendix S1. [file EAP-36-e70240-s001.pdf]

Appendix S1

Large-scale experimental evaluation of woody plant removal in desert grassland: Restoration, novelty, or degradation?

Brandon T. Bestelmeyer, Laura M. Burkett, Darren James, Juan Gamon, Robert L. Schooley

Ecological Applications.

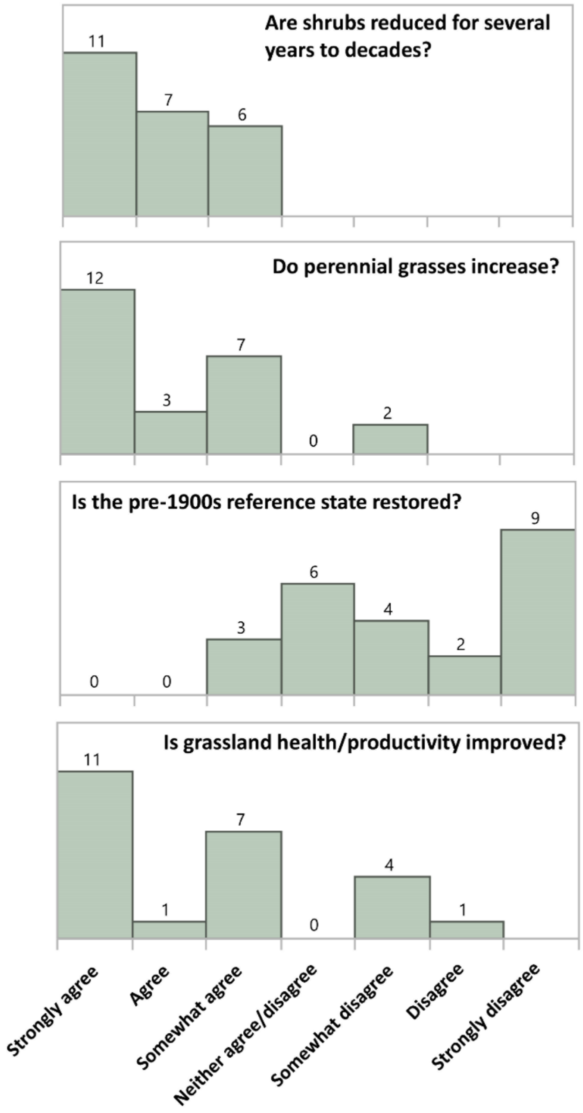

Figure S1. Results of a survey carried out in 2014 on beliefs of 24 Bureau of Land Management staff about the effects of woody plant removal treatments under the Restore New Mexico program in which responses to questions were recorded on the Likert scale. Survey was conducted electronically and included staff involved in rangeland and wildlife management.

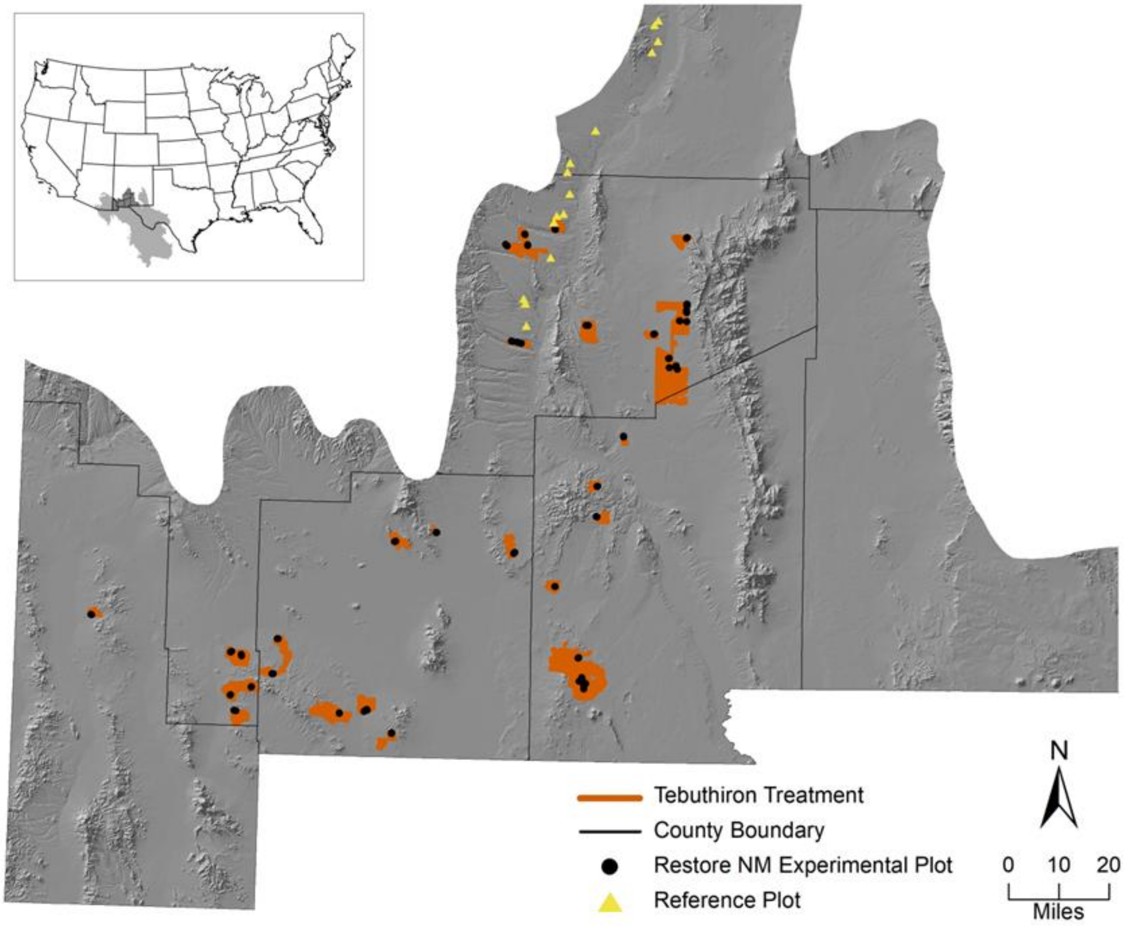

Figure S2. Locations of experimental plot pairs (dots represent adjacent control and treated plots) within woody plant removal areas in the Chihuahuan desert grassland region (see inset) of southwestern New Mexico, USA. Reference plots (yellow triangles) are also indicated.

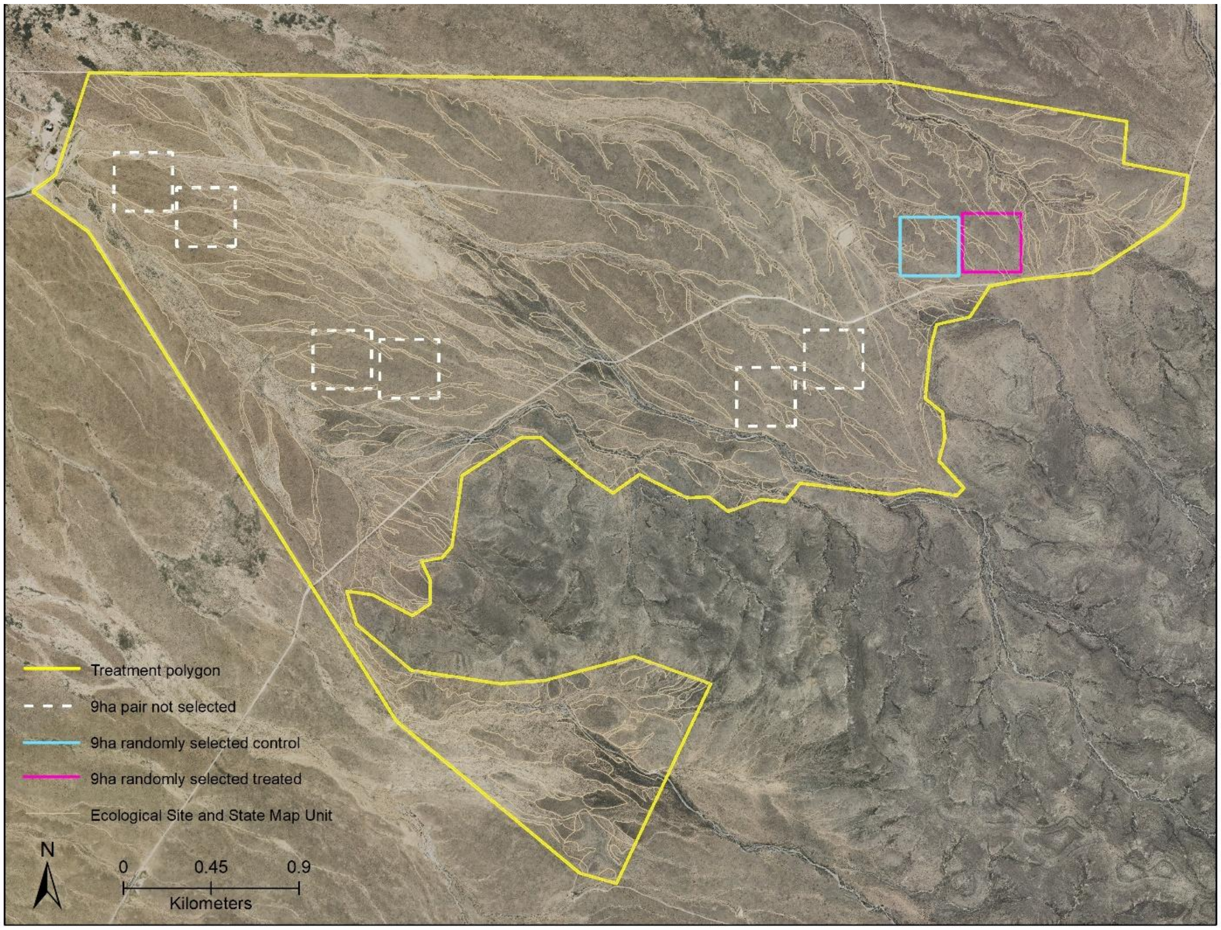

Figure S3. Example of the procedure to select plot pairs within a woody plant removal treatment polygon. Multiple candidate plot pairs where soils and vegetation composition could be matched between adjacent 9 ha areas were created in a GIS, based on soil and topographic data (ecological site maps and Digital Elevation Models) and vegetation state assessed using high resolution (1 m) aerial photographs (USDA National Agriculture Imagery Program). One or more pairs was then randomly selected to represent the treated area (colored squares) and one the plot pairs was randomly assigned to be the control (blue square).

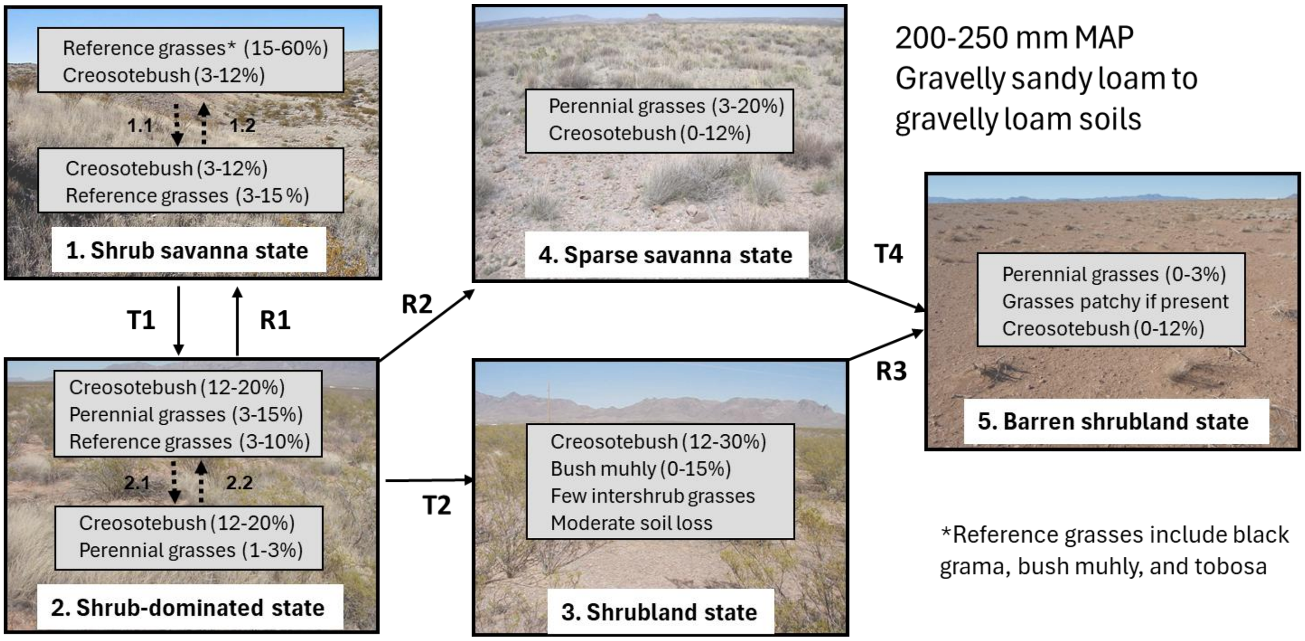

T1. Continuous heavy grazing, thinning and patchy loss of reference grasses, shrub proliferation
R1. Shrub control associated with grazing deferment or prescribed grazing and climate permitting grass recovery
R2. Shrub control after grass is sparse or followed by poorly planned grazing
T2. Loss of remaining interspace grasses, gradual loss of soil organic matter, infill of shrubs, and soil erosion
R3. Shrub control after soil loss is significant, precluding substantial grass recovery
T4. Poorly planned grazing or regional increases in aridity

Figure S4. A state-and-transition model for the primary soil and vegetation setting within which woody plant removal treatments were applied. This model is used by US government agencies as part of rangeland health assessments, to guide management decisions and as a tool for engagement with ranchers. MAP= mean annual precipitation. Black grama =*Bouteloua eriopoda*, bush muhly = *Muhlenbergia porteri*, tobosa = *Pleuraphis mutica,* and creosotebush = *Larrea tridentata*. The model is housed online at https://edit.jornada.nmsu.edu/catalogs/esg/042B/ESG5. All background photographs were taken by Brandon T. Bestelmeyer.

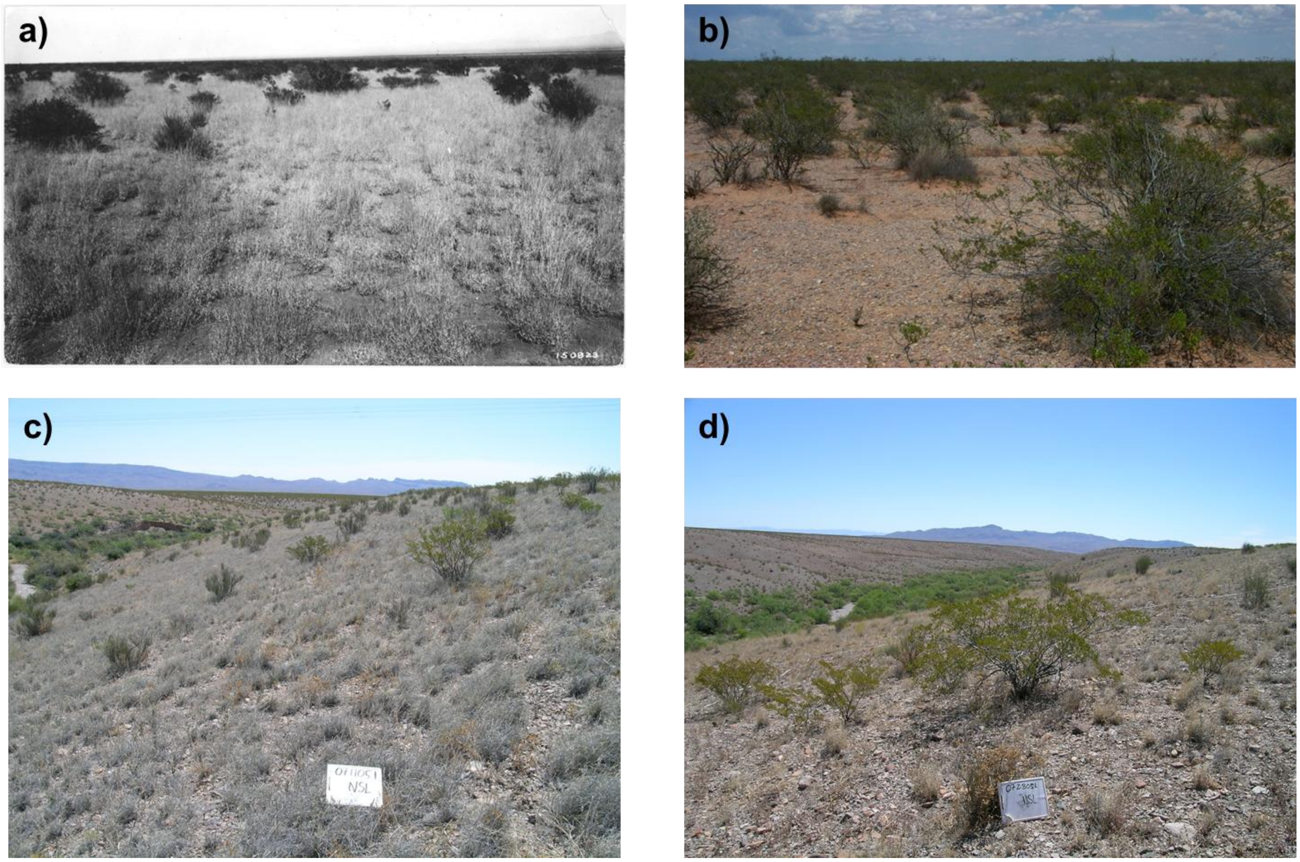

Figure S5. Examples of photographs used in defining reference sites. a) A reference savanna state with a high cover of reference perennial grass (black grama) and sparse creosotebush cover from the Jornada Experimental Range (32°29'13.34"N, 106°45'1.75"W) in 1920 at an elevation of 1330 m, which is close to the lowest elevations in our experimental samples (see Figure S6); b) the same location as a) in 2001, illustrating loss of grass cover and increase in bare ground and creosotebush cover; c) a reference site with relatively high cover of reference perennial grass (54.5%) at an elevation of 1477 m; d) a reference site with comparatively low cover of reference grasses (7.5%), likely due to management, but with well-distributed grass plants and low creosotebush cover at an elevation of 1391 m. Together c) and d) span the range of variation in reference grass cover that represent our reference sites. Photographic credits: a) and b), USDA-ARS Jornada Experimental Range; c) and d), Laura M. Burkett.

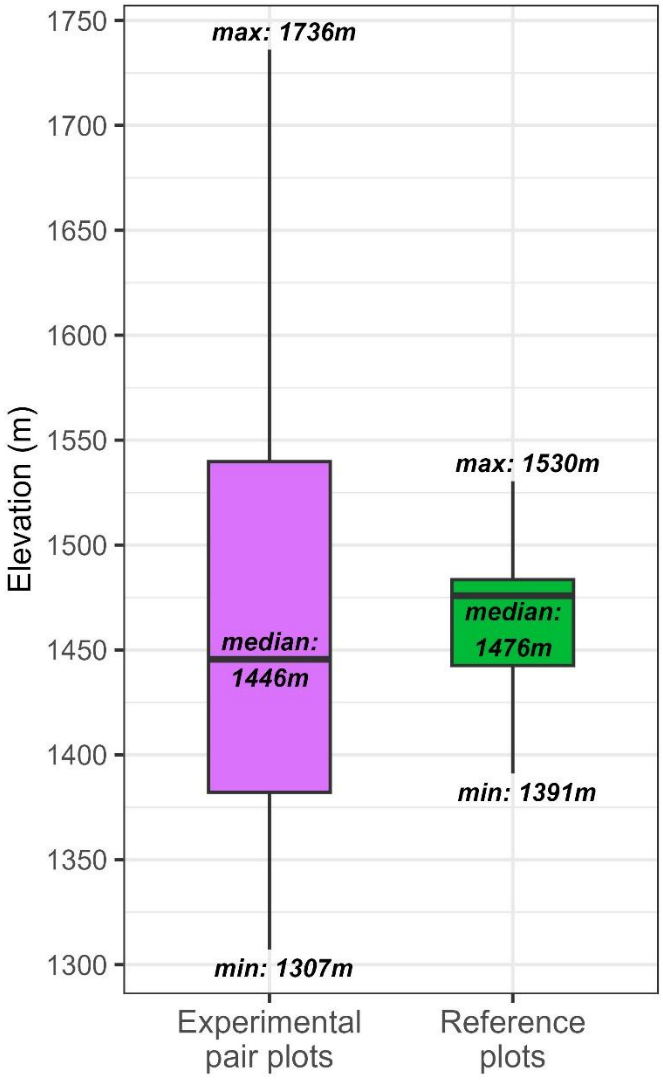

Figure S6. Elevation ranges for experimental plot pairs and reference plots measured in this study.

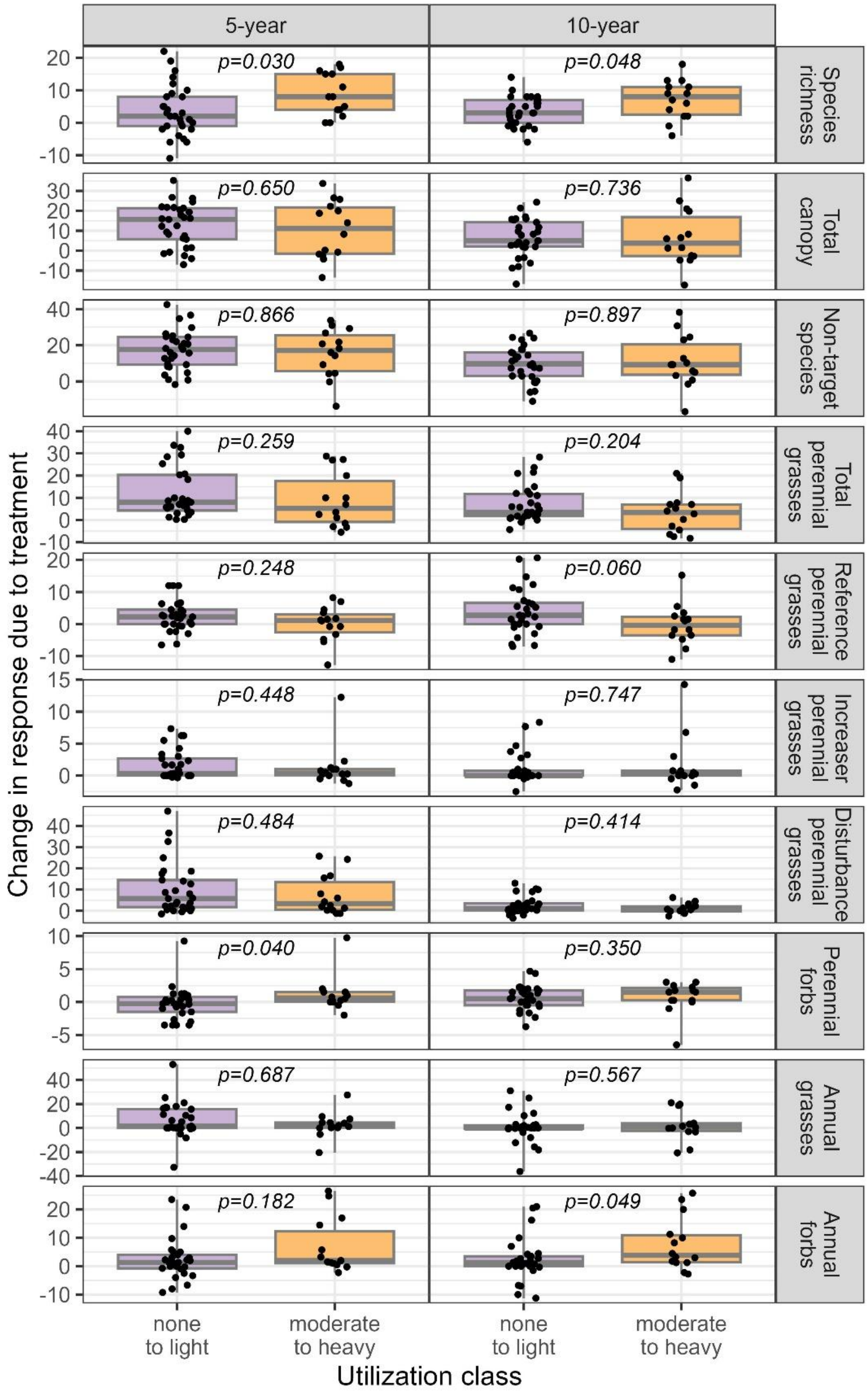

Figure S7. Effects of utilization class on gain scores for species richness and plant functional groups with Kruskal-Wallis p values.

Table S1. Criteria for generating and assigning species to vegetation indicators. Indicators were calculated as an average of all transects for a plot and a given year. Transect foliar cover for a given species group = 100% x the total number of points where a plant in the species group is intercepted by a pin drop divided by the total number of points on a transect (Herrick et al. 2017). See Appendix S2 for species assignments to indicators used in this paper.

**Indicator**	**Definition**	**How species were assigned**
Species richness	Number of species detected.	Each plant code is considered as one species.
Total canopy	Percent total foliar canopy cover (total canopy) is the proportion of the soil surface that is protected by the vertical projection of any plant part. Foliar canopy cover does not include litter, rocks, or biological soil crusts; nor does it include 'gaps' within the perimeter of a plant's canopy.	Foliar canopy cover includes any rooted plant part, live or dead. All plant species codes are included as total foliar canopy cover.
Non-target species	Percent foliar canopy cover of the non-target species group.	Non-target species group includes all species not targeted for removal by land managers. Non-target species group does not include encroaching shrubs.
Total perennial grasses	Percent total foliar cover of perennial grasses.	Perennial grass species group included all species classified as perennial graminoids using the PLANTS database (USDA Natural Resources Conservation Service 2023b). When PLANTS database listed multiple durations for a grass species, we considered information in Allred (2016).
Reference perennial grasses	Percent total foliar cover of reference perennial grasses. Reference perennial grasses are long-lived perennial grass species indicative of, and dominant in, the reference state.	Reference perennial grasses were obtained from the state-and-transition models and ecological dynamics narratives of the relevant ecological site descriptions (USDA Natural Resources Conservation Service 2023a) within Major Land Resource Area (MLRA) codes 38X, 39X, 41X, 42A, 42B, and 42C in southern New Mexico.

Increaser perennial grasses	Percent total foliar cover of increaser perennial grasses. Increaser perennial grass species are persistent perennial grasses typically present, but not dominant, in the reference state that persist or increase in a plant community as reference perennial grasses decrease.	Increaser perennial grasses are any perennial grass species that do not belong to the reference perennial grass or disturbance perennial grass species groups. This group of grasses tends to increase in response to heavy grazing pressure (Allison and Ashcroft 2011).
Disturbance perennial grasses	Percent total foliar cover of disturbance perennial grasses. Disturbance perennial grass species are perennial grasses that increase in high precipitation years or after a disturbance. These are typically short-lived/shallow-rooted or invasive.	Disturbance perennial grass species were obtained from the state-and-transition models and ecological dynamics narratives of the relevant ecological site descriptions within MLRA codes 38X, 39X, 41X, 42A, 42B, and 42C in southern New Mexico, as well as from SEINET data (SEINet - AZ/NM Node 2023) and local knowledge.
Perennial forbs	Percent total foliar cover of perennial forbs.	Perennial forb species group included all species classified as perennial forbs/herbs by the PLANTS database. When the PLANTS database listed multiple durations or lifeforms, then we considered Allred (2023) and when that was not definitive, we used local knowledge.
Annual grasses	Percent total foliar cover of annual grasses.	Annual grass species group included all species classified as annual graminoids by the PLANTS database. When PLANTS database listed multiple durations for a grass species, Allred (2016) was used.
Annual forbs	Percent total foliar cover of annual forbs.	Annual forbs species group included all species classified as annual forbs/herbs by the PLANTS database. When the PLANTS database listed multiple durations or lifeforms, then we considered Allred (2023) and when that was not definitive, we used local knowledge.
All shrubs	Percent total foliar cover of shrubs.	Shrub species group included all species classified as shrubs by the PLANTS database. When the PLANTS database listed lifeforms,

		then we considered Allred (2023) and when that was not definitive, we used local knowledge. Sub-shrubs were not included in this species group.
Encroaching shrubs	Percent total foliar cover of encroaching (target) shrubs	Includes shrub species targeted for shrub removal treatment (creosotebush and tarbush).

References

Allison, C. B., and N. Ashcroft. 2011. New Mexico range plants, Circular 374. New Mexico State University.

Allred, K. W. 2016. A field guide to the grasses of New Mexico, 3rd edition. Agricultural Experiment Station, New Mexico State University, Las Cruces, NM.

Allred, K. W. 2023. Pocket guide to the flora of the Jornada Plain, 9th edition. Kelly W. Allred, Las Cruces, NM.

Herrick, J. E., J. W. V. Zee, S. E. McCord, E. M. Courtright, J. W. Karl, and L. M. Burkett. 2017. Monitoring manual for grassland, shrubland and savanna ecosystems. 2nd Ed. Volume I: Core Methods. USDA-ARS Jornada Experimental Range, Las Cruces, NM, USA.

SEINet - AZ/NM Node. 2023. Biodiversity occurrence data. https://swbiodiversity.org/seinet/index.php.

USDA Natural Resources Conservation Service. 2023a. Ecosystem Dynamics Interpretative Tool. https://edit.jornada.nmsu.edu/.

USDA Natural Resources Conservation Service. 2023b. PLANTS Database. https://plants.usda.gov.

Table S2. Definitions and source data for non-climate predictor variables used to model shrub removal effects.

Predictor Variable	Definition	Data Source
Elevation	Plot pair mean elevation, in feet.	Field data acquired from Garmin GPSMap 64sx or similar.
Slope	Plot pair mean slope.	Field data acquired using a clinometer.
Maximum % clay	Plot pair mean maximum percent clay encountered within 50 cm of the soil surface or to root restrictive horizons if shallower than 50 cm.	Field data; hand texture percent clay was estimated by horizon.
Encroaching shrub cover	Gain score of the encroaching shrub percent foliar canopy cover.	Field data acquired using line-point intercept.
Baseline cover	Plot pair mean baseline cover value for response indicator of interest.	Field data acquired using line-point intercept.

Table S3. Candidate precipitation (PPT) predictor variables used to model effects of woody plant removal. For each plant functional group response, the best performing (lowest AICc value) candidate variable was used alongside variables in Table S1 in generalized linear models. Annual PPT for a given year is the sum of PPT for November 1 of the prior year through October 31 of the current year. Growing season PPT is the sum of PPT of July, August, and September for a given year. Climate normals (three decade average PPT) were for 1994-2024. All PPT variables were calculated from monthly gridded (4km) PRISM data (PRISM Climate Group, Oregon State University, https://prism.oregonstate.edu, data created 2 Jan 2025, accessed 7 Jan 2025).

**Candidate PRISM Climate Variable**	**Calculation/Formula**
Deviation of prior year annual precipitation from 30-year normal (PY)	PY = [(30y water year PPT) – (water year PPT prior to the sampling year)]/2.
Deviation of same year annual precipitation difference from the 30-year (SY)	SY = [(30y water year PPT) – (water year PPT for the sampling year)]/2.
Average deviation of prior year and same year annual precipitation difference from 30-year normal (PYSY)	PYSY= (PY+ SY) / 2
Deviation of prior year growing season precipitation from 30-year normal (PYGS)	PYGS = [(30Y growing season PPT) – (growing season PPT for the year prior to the sampling year)]/2.
Deviation of same year growing season precipitation from 30-year normal (SYGS)	SYGS = [(30Y growing season PPT)-(sampling year growing season PPT)]/2.
Average deviation of the prior year and same year growing season precipitation from 30-year normal (PYSYGS)	PYSYGS = (PYGS+ SYGS)/2

Table S4. Best precipitation variable used in models of effects of woody plant removal for plant functional groups in Figure 5. See code definitions in Table S2.

**Indicator**	**5-year gain scores**	**10-year gain scores**
Total canopy cover	SYGS	PYSYGS
Non-target species	PYGS	PYSYGS
Total perennial grasses	SYGS	intercept only
Reference grasses	PYGS	intercept only
Increaser perennial grasses	intercept only	intercept only
Disturbance perennial grasses	SYGS	PYSY
Annual grasses	intercept only	PYSYGS
Annual forbs	PY	PYGS

Table S5. Results of Type III tests of fixed effects for the 15-year comparisons of the cover of key plant functional groups in treatment and control plots (see Figure 2). P values < 0.05 in bold.

**Indicator**	**Effect**	**NumDF**	**DenDF**	***F***	***P***
Total canopy	treatment	1	8.36	8.42	**0.0190**
	year	3	16	44.67	**<0.0001**
	treatment * year	3	16	7.26	**0.0027**
Total perennial grasses	treatment	1	7.92	11.6	**0.0094**
	year	3	16	36.87	**<0.0001**
	treatment * year	3	16	10.83	**0.0004**
Reference grasses	treatment	1	10.6	2.83	0.1217
	year	3	16	12.46	**0.0002**
	treatment * year	3	16	0.56	0.6506
Encroaching shrubs	treatment	1	18	27.6	**<.0001**
	year	3	16	13.92	**0.0001**
	treatment * year	3	16	3.52	**0.0394**
