# Supplementary material for: Large‐scale experimental evaluation of woody plant removal in desert grassland: Restoration, novelty, or degradation?
Source: Ecol Appl. 2026 Apr 21;36:e70240. doi: 10.1002/eap.70240 (PMC13099281; doi:10.1002/eap.70240)
Supplement: Supplementary file 2 — Appendix S2. [file EAP-36-e70240-s002.pdf]

Appendix S2

Large-scale experimental evaluation of woody plant removal in desert grassland: Restoration, novelty, or degradation?

Brandon T. Bestelmeyer, Laura M. Burkett, Darren James, Juan Gamon, Robert L. Schooley

Ecological Applications.

Table S1. Assignment of plant species to plant functional groups used in analyses.

Species Code	Scientific Name	Duration	Subcategory	Plant Functional Group	Non-target /Encroaching Shrub Species Group	Perennial Grass Functional Groups	MRPP Functional Group
ACNA2	*Acourtia nana*	Perennial	Forb	Perennial forb	Non-target	·	Perennial forb
AF01	unknown annual forb species 1	Annual	Forb	Annual forb	Non-target	·	Annual forb
AF02	unknown annual forb species 2	Annual	Forb	Annual forb	Non-target	·	Annual forb
AF03	unknown annual forb species 3	Annual	Forb	Annual forb	Non-target	·	Annual forb
AF04	unknown annual forb species 4	Annual	Forb	Annual forb	Non-target	·	Annual forb
AF05	unknown annual forb species 5	Annual	Forb	Annual forb	Non-target	·	Annual forb
AF06	unknown annual forb species 6	Annual	Forb	Annual forb	Non-target	·	Annual forb
AF33	unknown annual forb species 33	Annual	Forb	Annual forb	Non-target	·	Annual forb
AF34	unknown annual forb species 34	Annual	Forb	Annual forb	Non-target	·	Annual forb
AG01	unknown annual grass species 1	Annual	Grass	Annual grass	Non-target	·	Annual grass
AG02	unknown annual grass species 2	Annual	Grass	Annual grass	Non-target	·	Annual grass
AG03	unknown annual grass species 3	Annual	Grass	Annual grass	Non-target	·	Annual grass
AG04	unknown annual grass species 4	Annual	Grass	Annual grass	Non-target	·	Annual grass
AG05	Unknown annual *Panicum*	Annual	Grass	Annual grass	Non-target	·	Annual grass
AG06	Unknown annual *Bouteloua*	Annual	Grass	Annual grass	Non-target	·	Annual grass
ALIN	*Allionia incarnata*	Annual	Forb	Annual forb	Non-target	·	Annual forb
ALLIU	*Allium* species	Perennial	Forb	Perennial forb	Non-target	·	Perennial forb
ALWR	*Aloysia wrightii*	Perennial	Shrub	Shrub	Non-target	·	·
AMARA	*Amaranthus* species	Annual	Forb	Annual forb	Non-target	·	Annual forb
AMBRO	*Ambrosia* species	Annual	Forb	Annual forb	Non-target	·	Annual forb
ARAD	*Aristida adscensionis*	Annual	Grass	Annual grass	Non-target	·	Annual grass
ARIST	*Aristida* species	Perennial	Grass	Perennial grass	Non-target	Increaser	Perennial bunchgrass

ARPA9	*Aristida pansa*	Perennial	Grass	Perennial grass	Non-target	Increaser	Perennial bunchgrass
ARPU9	*Aristida purpurea*	Perennial	Grass	Perennial grass	Non-target	Increaser	Perennial bunchgrass
ARTE3	*Aristida ternipes*	Perennial	Grass	Perennial grass	Non-target	Increaser	Perennial bunchgrass
ASAL5	*Astragalus albulus*	Perennial	Forb	Perennial forb	Non-target	·	Perennial forb
ASTRA	*Astragalus* species	Annual	Forb	Annual forb	Non-target	·	Annual forb
ATCA2	*Atriplex canescens*	Perennial	Shrub	Shrub	Non-target	·	·
BAAB	*Bahia absinthifolia*	Perennial	Forb	Perennial forb	Non-target	·	Perennial forb
BACCH	*Baccharis* species	Perennial	Shrub	Shrub	Non-target	·	·
BAMU	*Baileya multiradiata*	Perennial	Forb	Perennial forb	Non-target	·	Perennial forb
BOAR	*Bouteloua aristidoides*	Annual	Grass	Annual grass	Non-target	·	Annual grass
BOBA2	*Bouteloua barbata*	Annual	Grass	Annual grass	Non-target	·	Annual grass
BOBA3	*Bothriochloa barbinodis*	Perennial	Grass	Perennial grass	Non-target	Increaser	Perennial bunchgrass
BOCU	*Bouteloua curtipendula*	Perennial	Grass	Perennial grass	Non-target	Reference	Reference grass
BOER4	*Bouteloua eriopoda*	Perennial	Grass	Perennial grass	Non-target	Reference	Reference grass
BOERH2	*Boerhavia* species	Annual	Forb	Annual forb	Non-target	·	Annual forb
BOGR2	*Bouteloua gracilis*	Perennial	Grass	Perennial grass	Non-target	Reference	Reference grass
BOLA2	*Bothriochloa laguroides*	Perennial	Grass	Perennial grass	Non-target	Increaser	Perennial bunchgrass
BOTHR	*Bothriochloa* species	Perennial	Grass	Perennial grass	Non-target	Increaser	Perennial bunchgrass
CAER	*Calliandra eriophylla*	Perennial	Shrub	Shrub	Non-target	·	·
CHAMA15	*Chamaesyce* species	Annual	Forb	Annual forb	Non-target	·	Annual forb
CHENO	*Chenopodium* species	Annual	Forb	Annual forb	Non-target	·	Annual forb
CHER2	*Chaetopappa ericoides*	Perennial	Forb	Perennial forb	Non-target	·	Perennial forb
CHIN2	*Chenopodium incanum*	Annual	Forb	Annual forb	Non-target	·	Annual forb
CHLA10	*Chamaesyce lata*	Perennial	Forb	Perennial forb	Non-target	·	Perennial forb
CHSO	*Chamaesaracha sordida*	Perennial	Forb	Perennial forb	Non-target	·	Perennial forb
CHVI4	*Chloris virgata*	Annual	Grass	Annual grass	Non-target	·	Annual grass
COMA14	*Coryphantha macromeris*	Perennial	Succulent	·	Non-target	·	Succulent
CRCR3	*Cryptantha crassisepala*	Annual	Forb	Annual forb	Non-target	·	Annual forb

CRPO5	*Croton pottsii*	Perennial	Forb	Perennial forb	Non-target	·	Perennial forb
CRYPT	*Cryptantha* species	Annual	Forb	Annual forb	Non-target	·	Annual forb
CYIM2	*Cylindropuntia imbricata*	Perennial	Succulent	·	Non-target	·	Succulent
CYLE8	*Cylindropuntia leptocaulis*	Perennial	Succulent	·	Non-target	·	Succulent
CYLIN2	*Cylindropuntia* species	Perennial	Succulent	·	Non-target	·	Succulent
DABR	*Dalea brachystachya*	Annual	Forb	Annual forb	Non-target	·	Annual forb
DAFO	*Dalea formosa*	Perennial	Sub-Shrub	·	Non-target	·	Sub-Shrub
DAPU7	*Dasyochloa pulchella*	Perennial	Grass	Perennial grass	Non-target	Disturbance	Fluffgrass
DEPI	*Descurainia pinnata*	Annual	Forb	Annual forb	Non-target	·	Annual forb
DIWI2	*Dimorphocarpa wislizeni*	Annual	Forb	Annual forb	Non-target	·	Annual forb
DRCU	*Draba cuneifolia*	Annual	Forb	Annual forb	Non-target	·	Annual forb
ENDE	*Enneapogon desvauxii*	Perennial	Grass	Perennial grass	Non-target	Disturbance	Other perennial grass
EPHED	*Ephedra* species	Perennial	Shrub	Shrub	Non-target	·	·
EPTO	*Ephedra torreyana*	Perennial	Shrub	Shrub	Non-target	·	·
EPTR	*Ephedra trifurca*	Perennial	Shrub	Shrub	Non-target	·	·
ERAB2	*Eriogonum abertianum*	Annual	Forb	Annual forb	Non-target	·	Annual forb
ERAGR	*Eragrostis* species	Perennial	Grass	Perennial grass	Non-target	Disturbance	Perennial bunchgrass
ERCI	*Eragrostis cilianensis*	Annual	Grass	Annual grass	Non-target	·	Annual grass
ERIOG	*Eriogonum* species	Annual	Forb	Annual forb	Non-target	·	Annual forb
ERLE	*Eragrostis lehmanniana*	Perennial	Grass	Perennial grass	Non-target	Disturbance	Perennial bunchgrass
ERPE	*Eragrostis pectinacea*	Annual	Grass	Annual grass	Non-target	·	Annual grass
ERTR8	*Eriogonum trichopes*	Annual	Forb	Annual forb	Non-target	·	Annual forb
ERWR	*Eriogonum wrightii*	Perennial	Sub-Shrub	·	Non-target	·	Sub-Shrub
EUEX4	*Euphorbia exstipulata*	Annual	Forb	Annual forb	Non-target	·	Annual forb
FLCE	*Flourensia cernua*	Perennial	Shrub	Shrub	Encroaching shrub	·	·
GUMI	*Gutierrezia microcephala*	Perennial	Sub-Shrub	·	Non-target	·	Sub-Shrub
GUSA2	*Gutierrezia sarothrae*	Perennial	Sub-Shrub	·	Non-target	·	Sub-Shrub
GUSP	*Gutierrezia sphaerocephala*	Annual	Forb	Annual forb	Non-target	·	Annual forb

HECO26	*Hesperostipa comata*	Perennial	Grass	Perennial grass	Non-target	Reference	Reference grass
HODR	*Hoffmannseggia drepanocarpa*	Perennial	Forb	Perennial forb	Non-target	·	Perennial forb
HOFFM	*Hoffmannseggia* species	Perennial	Forb	Perennial forb	Non-target	·	Perennial forb
HOGL2	*Hoffmannseggia glauca*	Perennial	Forb	Perennial forb	Non-target	·	Perennial forb
IBTE2	*Ibervillea tenuisecta*	Perennial	Forb	Perennial forb	Non-target	·	Perennial forb
IPOMO	*Ipomoea* species	Annual	Forb	Annual forb	Non-target	·	Annual forb
ISTE2	*Isocoma tenuisecta*	Perennial	Sub-Shrub	·	Non-target	·	Sub-Shrub
KALLS	*Kallstroemia* species	Annual	Forb	Annual forb	Non-target	·	Annual forb
KAPA	*Kallstroemia parviflora*	Annual	Forb	Annual forb	Non-target	·	Annual forb
KOSP	*Koeberlinia spinosa*	Perennial	Shrub	Shrub	Non-target	·	·
KRER	*Krameria erecta*	Perennial	Sub-Shrub	·	Non-target	·	Sub-Shrub
KRLA	*Krameria lanceolata*	Perennial	Forb	Perennial forb	Non-target	·	Perennial forb
KRLA2	*Krascheninnikovia lanata*	Perennial	Sub-Shrub	·	Non-target	·	Sub-Shrub
LAOC3	*Lappula occidentalis*	Annual	Forb	Annual forb	Non-target	·	Annual forb
LATR2	*Larrea tridentata*	Perennial	Shrub	Shrub	Encroaching shrub	·	·
LEFE	*Lesquerella fendleri*	Perennial	Forb	Perennial forb	Non-target	·	Perennial forb
LEGO	*Lesquerella gordonii*	Perennial	Forb	Perennial forb	Non-target	·	Perennial forb
LESQU	*Lesquerella* species	Perennial	Forb	Perennial forb	Non-target	·	Perennial forb
LINUM	*Linum* species	Annual	Forb	Annual forb	Non-target	·	Annual forb
LYBE	*Lycium berlandieri*	Perennial	Shrub	Shrub	Non-target	·	·
LYCIU	*Lycium* species	Perennial	Shrub	Shrub	Non-target	·	·
LYPA	*Lycium pallidum*	Perennial	Shrub	Shrub	Non-target	·	·
MATA2	*Machaeranthera tanacetifolia*	Annual	Forb	Annual forb	Non-target	·	Annual forb
MELE2	*Melampodium leucanthum*	Perennial	Forb	Perennial forb	Non-target	·	Perennial forb
MESC	*Menodora scabra*	Perennial	Forb	Perennial forb	Non-target	·	Perennial forb
MOCE	*Mollugo cerviana*	Annual	Forb	Annual forb	Non-target	·	Annual forb
MUAR	*Muhlenbergia arenacea*	Perennial	Grass	Perennial grass	Non-target	Reference	Reference grass
MUAR2	*Muhlenbergia arenicola*	Perennial	Grass	Perennial grass	Non-target	Increaser	Perennial bunchgrass

MUPO2	*Muhlenbergia porteri*	Perennial	Grass	Perennial grass	Non-target	Reference	Reference grass
MUSQ3	*Munroa squarrosa*	Annual	Grass	Annual grass	Non-target	·	Annual grass
MUTO2	*Muhlenbergia torreyi*	Perennial	Grass	Perennial grass	Non-target	Increaser	Perennial bunchgrass
OPEN3	*Opuntia engelmannii*	Perennial	Succulent	·	Non-target	·	Succulent
OPMA8	*Opuntia macrocentra*	Perennial	Succulent	·	Non-target	·	Succulent
OPPO	*Opuntia polyacantha*	Perennial	Succulent	·	Non-target	·	Succulent
OPUNT	*Opuntia* species	Perennial	Succulent	·	Non-target	·	Succulent
PAHI5	*Panicum hirticaule*	Annual	Grass	Annual grass	Non-target	·	Annual grass
PAIN2	*Parthenium incanum*	Perennial	Sub-Shrub	·	Non-target	·	Sub-Shrub
PAOB	*Panicum obtusum*	Perennial	Grass	Perennial grass	Non-target	Reference	Reference grass
PEAN	*Pectis angustifolia*	Annual	Forb	Annual forb	Non-target	·	Annual forb
PF01	unknown perennial forb species 1	Perennial	Forb	Perennial forb	Non-target	·	Perennial forb
PF02	unknown perennial forb species 2	Perennial	Forb	Perennial forb	Non-target	·	Perennial forb
PF03	unknown perennial forb species 3	Perennial	Forb	Perennial forb	Non-target	·	Perennial forb
PF05	unknown perennial forb species 5	Perennial	Forb	Perennial forb	Non-target	·	Perennial forb
PG01	unknown perennial grass species 1	Perennial	Grass	Perennial grass	Non-target	OtherPG	Other perennial grass
PG02	unknown perennial grass species 2	Perennial	Grass	Perennial grass	Non-target	OtherPG	Other perennial grass
PHAU13	*Phemeranthus aurantiacus*	Perennial	Forb	Perennial forb	Non-target	·	Perennial forb
PHIN	*Phacelia integrifolia*	Annual	Forb	Annual forb	Non-target	·	Annual forb
PLMU3	*Pleuraphis mutica*	Perennial	Grass	Perennial grass	Non-target	Reference	Reference grass
PLPA2	*Plantago patagonica*	Annual	Forb	Annual forb	Non-target	·	Annual forb
POAL4	*Polygala alba*	Perennial	Forb	Perennial forb	Non-target	·	Perennial forb
POLYG	*Polygala* species	Perennial	Forb	Perennial forb	Non-target	·	Perennial forb
POOL	*Portulaca oleracea*	Annual	Forb	Annual forb	Non-target	·	Annual forb
PORTU	*Portulaca* species	Annual	Forb	Annual forb	Non-target	·	Annual forb

PRGL2	*Prosopis glandulosa*	Perennial	Shrub	Shrub	·	·	·
PROBO	*Proboscidea* species	Annual	Forb	Annual forb	Non-target	·	Annual forb
RHMI3	*Rhus microphylla*	Perennial	Shrub	Shrub	Non-target	·	·
RHUS	*Rhus* species	Perennial	Shrub	Shrub	Non-target	·	·
SAAB	*Sanvitalia abertii*	Annual	Forb	Annual forb	Non-target	·	Annual forb
SATR12	*Salsola tragus*	Annual	Forb	Annual forb	Non-target	·	Annual forb
SCAR	*Schismus arabicus*	Annual	Grass	Annual grass	Non-target	·	Annual grass
SCBR2	*Scleropogon brevifolius*	Perennial	Grass	Perennial grass	Non-target	Increaser	Other perennial grass
SEBA3	*Senna bauhinioides*	Perennial	Forb	Perennial forb	Non-target	·	Perennial forb
SEFL3	*Senecio flaccidus*	Perennial	Forb	Perennial forb	Non-target	·	Perennial forb
SELE6	*Setaria leucopila*	Perennial	Grass	Perennial grass	Non-target	Increaser	Perennial bunchgrass
SEVI4	*Setaria viridis*	Annual	Grass	Annual grass	Non-target	·	Annual grass
SH01	unknown shrub species 1	Perennial	Shrub	Shrub	·	·	·
SH02	unknown shrub species 2	Perennial	Shrub	Shrub	·	·	·
SIAB	*Sida abutifolia*	Perennial	Forb	Perennial forb	Non-target	·	Perennial forb
SOEL	*Solanum elaeagnifolium*	Perennial	Forb	Perennial forb	Non-target	·	Perennial forb
SPCO4	*Sporobolus contractus*	Perennial	Grass	Perennial grass	Non-target	Increaser	Perennial bunchgrass
SPCR	*Sporobolus cryptandrus*	Perennial	Grass	Perennial grass	Non-target	Increaser	Perennial bunchgrass
SPHA	*Sphaeralcea hastulata*	Perennial	Forb	Perennial forb	Non-target	·	Perennial forb
SPHAE	*Sphaeralcea* species	Perennial	Forb	Perennial forb	Non-target	·	Perennial forb
SPORO	*Sporobolus* species	Perennial	Grass	Perennial grass	Non-target	Increaser	Perennial bunchgrass
SS01	unknown subshrub species 1	Perennial	Sub-Shrub	·	Non-target	·	Sub-Shrub
SS02	unknown subshrub species 2	Perennial	Sub-Shrub	·	Non-target	·	Sub-Shrub
TARA	*Tamarix ramosissima*	Perennial	Tree	·	Non-target	·	·
THAC	*Thymophylla acerosa*	Perennial	Sub-Shrub	·	Non-target	·	Sub-Shrub
THPE4	*Thymophylla pentachaeta*	Perennial	Forb	Perennial forb	Non-target	·	Perennial forb
THYMO	*Thymophylla* species	Perennial	Forb	Perennial forb	Non-target	·	Perennial forb
TICA3	*Tiquilia canescens*	Perennial	Sub-Shrub	·	Non-target	·	Sub-Shrub

TILA2	*Tidestromia lanuginosa*	Annual	Forb	Annual forb	Non-target	·	Annual forb
TRMU	*Tridens muticus*	Perennial	Grass	Perennial grass	Non-target	Reference	Reference grass
VEEN	*Verbesina encelioides*	Annual	Forb	Annual forb	Non-target	·	Annual forb
YUBA	*Yucca baccata*	Perennial	Succulent	·	Non-target	·	Succulent
YUEL	*Yucca elata*	Perennial	Succulent	·	Non-target	·	Succulent
ZIAC	*Zinnia acerosa*	Perennial	Sub-Shrub	·	Non-target	·	Sub-Shrub
ZIGR	*Zinnia grandiflora*	Perennial	Sub-Shrub	·	Non-target	·	Sub-Shrub
ZINNI	*Zinnia* species	Perennial	Sub-Shrub	·	Non-target	·	Sub-Shrub
